# Genomic and transcriptomic insights into the efficient entomopathogenicity of *Bacillus thuringiensis*

**DOI:** 10.1038/srep14129

**Published:** 2015-09-28

**Authors:** Lei Zhu, Donghai Peng, Yueying Wang, Weixing Ye, Jinshui Zheng, Changming Zhao, Dongmei Han, Ce Geng, Lifang Ruan, Jin He, Ziniu Yu, Ming Sun

**Affiliations:** 1 State Key Laboratory of Agricultural Microbiology, College of Life Science and Technology, Huazhong Agricultural University, Wuhan, Hubei, People’s Republic of China

## Abstract

*Bacillus thuringiensis* has been globally used as a microbial pesticide for over 70 years. However, information regarding its various adaptions and virulence factors and their roles in the entomopathogenic process remains limited. In this work, we present the complete genomes of two industrially patented *Bacillus thuringiensis* strains (HD-1 and YBT-1520). A comparative genomic analysis showed a larger and more complicated genome constitution that included novel insecticidal toxicity-related genes (ITRGs). All of the putative ITRGs were summarized according to the steps of infection. A comparative genomic analysis showed that highly toxic strains contained significantly more ITRGs, thereby providing additional strategies for infection, immune evasion, and cadaver utilization. Furthermore, a comparative transcriptomic analysis suggested that a high expression of these ITRGs was a key factor in efficient entomopathogenicity. We identified an active extra urease synthesis system in the highly toxic strains that may aid *B. thuringiensis* survival in insects (similar to previous results with well-known pathogens). Taken together, these results explain the efficient entomopathogenicity of *B. thuringiensis*. It provides novel insights into the strategies used by *B. thuringiensis* to resist and overcome host immune defenses and helps identify novel toxicity factors.

*Bacillus thuringiensis*, a member of the *Bacillus cereus sensu lato* group, is a natural insecticidal bacterium. The bacterium produces parasporal crystals when it sporulates in limited nutrient conditions. These crystals consist of crystal proteins (Cry proteins, also named delta-endotoxins) and exhibit insecticidal activity against a range of invertebrates, including members of the orders Lepidoptera, Diptera, Coleoptera, some nematodes, mites, and protozoa^1^,^2^. For over 70 years, *B. thuringiensis* products, which contain a mixture of spores and insecticidal crystals, have been the most important biopesticides in fields such as agriculture and health. Compared with chemical pesticides, they have the advantages of specificity and high efficiency and are environmentally safe^3^.

However, the thousands of *B. thuringiensis* isolated strains show considerable variety in their insecticidal toxicity and spectra. Only highly toxic strains are used for biopesticide production. *B. thuringiensis* serovar *kurstaki* HD-1 is the original strain used in the microbial insecticide Dipel. Since its isolation in 1970, the products from strain HD-1 and similar strains (*Btk*) have been used as effective biopesticides. Strain HD-1 has also been designated as the primary U.S. reference standard strain for toxicity evaluation in all commercial *B. thuringiensis* formulations. This strain has enjoyed the greatest commercial success for microbial control worldwide^4^.

To understand the mechanism of *B. thuringiensis* insecticidal toxicity, early research primarily focused on the pore-forming mechanism of the parasporal crystal proteins (Cry and Cyt proteins) in insect gut epithelial cells^5^. However, controversy remained regarding whether *B. thuringiensis* was a toxin-producing soil bacterium (such as *Streptomyces* species) or an insect pathogen^6^,^7^. Subsequently, studies demonstrated that in addition to its role as an efficient insecticidal microorganism, *B. thuringiensis* could also produce virulent, pathogenic, and adaption factors during the infection process^8^. These results suggested that *B. thuringiensis* possessed a complicated pathogenic mechanism. Due to the rapid development of high-throughput sequencing technology, many *B. thuringiensis* genomes have been completed in recent years^9^,^10^,^11^,^12^,^13^,^14^,^15^,^16^. However, with the exception of two draft genomes of *Btk*^17^,^18^, no complete genomes of the biopesticide production strains (especially *Btk* HD-1 and similar strains) are currently available. Furthermore, there is a lack of systematic explanations on the effective insecticidal toxicity phenotype of these *B. thuringiensis* strains at the genomic, transcriptomic, or omic levels.

In this study, we completed the genome sequence of *B. thuringiensis* insecticide standard strain HD-1 and the related strain YBT-1520, which is also widely used for *Bt* biopesticide production (product trademark “Mianfeng”) in China in an attempt to obtain a comprehensive understanding of *B. thuringiensis* pathogenesis. Additionally, we performed a comparative study with other *B. cereus* group genomes. A comparative genomic analysis revealed that the *Btk* strains possess a larger number and more types of insecticidal toxicity-related genes (ITRGs) compared with other *B. cereus* group bacteria with characteristic chromosomal features. Interestingly, the comparative transcriptomic analysis (a microarray of strain YBT-1520) indicated that the ITRGs in *Btk* are not only all actively transcribed but are also transcribed at a substantially higher level than general *B. thuringiensis*. This work explains the efficient entomopathogenicity of *B. thuringiensis*, especially regarding the increased transcription of ITRGs in highly toxic strains.

## Results

### General genome feature: larger genome size and more plasmids

The chromosome of strain HD-1 and strain YBT-1520 are circular molecules that are 5,631,672 bp and 5,602,565 bp in length, respectively, with the same average G + C content (35.3%) (Table 1). There were no significant differences between the chromosomes of strain HD-1 and strain YBT-1520; therefore, the genes discussed below should have common names. Strain HD-1 contained 13 plasmids with a combined size of 1,135 kb, whereas strain YBT-1520 contained 11 plasmids with a combined size of approximately 978 kb (Supplementary Table S1). Most of the plasmids had significantly lower G + C contents than the chromosome, with the exception of pBMB46 and pBMBLin15 (35.4% and 40.1%) in strain HD-1, which are a putative circular prophage and a linear prophage, respectively. The chromosome of strain HD-1 contained 5,864 genes, 41 rRNA genes, and 94 tRNA, whereas strain YBT-1520 contained 5,830 genes, 39 rRNA genes, and 98 tRNA (Table 1). The plasmids of strain HD-1 and strain YBT-1520 contained 1,064 and 890 genes, respectively.

**Figure 1:**
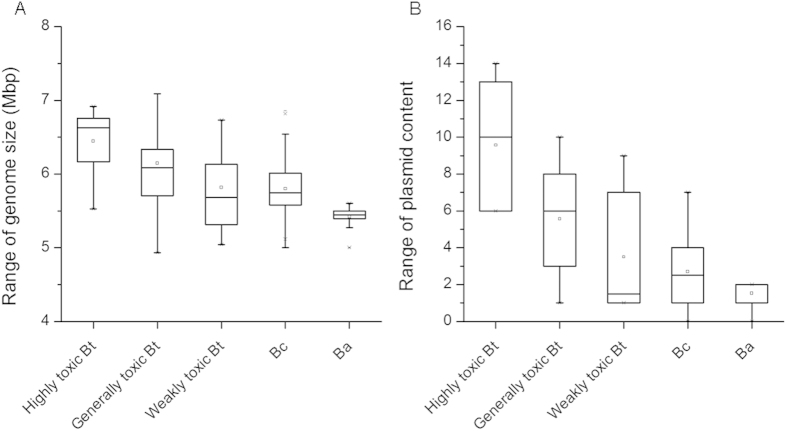
Comparison of the whole genome size and plasmid content of the *B. cereus* group strains. The 304 *B. cereus* group strains were downloaded from the NCBI genome database. The *B. thuringiensis* strains listed in Table 1 and Supplementary Table S2 were sorted on the basis of their insecticidal toxicity level from high to low.

Because the genome data available for the *B. cereus sensu lato* group in NCBI include 50 *B. thuringiensis*, 161 *B. cereus* and 93 *B. anthracis* strains annotated according to previously reported insecticidal activities and serotypes, we divided the 50 *B. thuringiensis* strains into three groups: highly, generally and weakly toxic strains (12, 29 and 9 strains, respectively, Table 1 and Supplementary Table S2). The comparison showed that the *B. thuringiensis* strains with high insecticidal toxicity^10^,^12^,^19^ had significantly larger genome sizes and were richer in plasmid content than the weakly toxic *B. thuringiensis* strains, and typical *B. cereus*, *B. anthracis* strains which are not toxic to insects (P < 0.05, T-test, Fig. 1A,B).

### A chromosomal inversion with novel insecticidal toxicity-related genes (ITRGs)

The genomes of the *B. cereus* group strains possessed high sequence homology, protein identity, and conserved synteny, with the exception of a laboratory *B. anthracis* mutant strain CDC684 that had a 3.3 Mb chromosomal inversion^20^. Compared with the other *B. cereus* group genomes, *B. thuringiensis* serovar *kurstaki* strains (including HD-1, YBT-1520, HD73^9^ and T03a001^21^) all had a massive and rare inversion with a 740 kb fragment on the chromosome relative to the replication origin.

**Figure 2:**
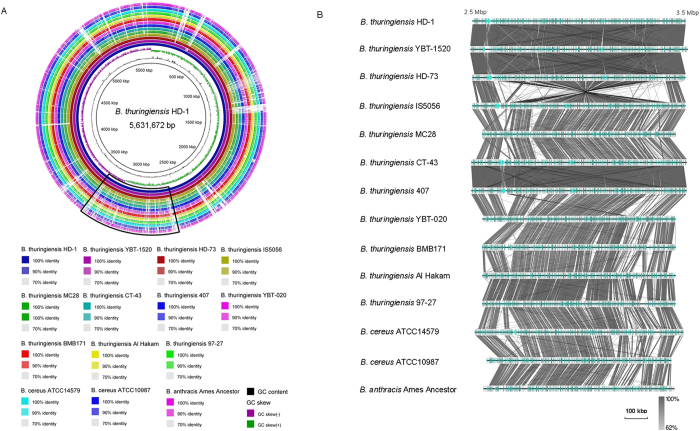
Chromosome alignments of *B. cereus* group members. (**A**) The diagram represents the BLASTn results of a circular chromosome comparison using the BRIG program^59^. Each genome is color-coded as indicated by the legend. Relative shading density (from darker to lighter) within each circle represents relative levels of nucleotide homology. White regions indicate regions with no identity to the reference. (**B**) Chromosome inversion region comparison. Genes are represented by colored arrows. Well-conserved segments of the chromosomes are paired using shaded regions; dark gray indicates 62 to 100% nucleotide identity. The non-shaded regions lack homology between the *B. cereus* group strains. The outer scale is given in kilobases.

The inversion in the HD-1 chromosome is located between the base-pair coordinates 2,644 kb and 3,380 kb; in the YBT-1520 chromosome, the inversion is located between 2,613 kb and 3,351 kb. In contrast to *B. anthracis* strain CDC684, the genome rearrangement events did not occur between homologous lambda-like prophage regions^20^ but instead occurred between two reversed IS232A elements (Fig. 2A,B). Interestingly, we found that most of the genes that flank the two IS232A regions are unique genes in the *Btk* strains, such as a novel HBL gene operon (*hblIII2*) and a putative pectate lyase family gene (*pel2*). Notably, *Btk* strain HD73, which is listed in the “general” toxicity group, has the same chromosomal inversion and these novel virulence related genes. It is a special case in *Btk* because that ,as previous work reported^9^, the “general” toxic strain HD73 only contains one parasporal crystal protein gene *cry1Ac*, and protein Cry1Ac is the primary toxin for the insecticidal phenotype. Additionally, a comparative analysis of the completed *B. cereus* group genomes showed a large number of functional genes in the chromosomal inversion region in the *Btk* strains, including amino acid transporters, various resistant proteins, and regulators, which were not highly conserved in the other strains (Fig. 2A).

### Highly toxic B. thuringiensis strains encode more ITRGs

**Figure 3:**
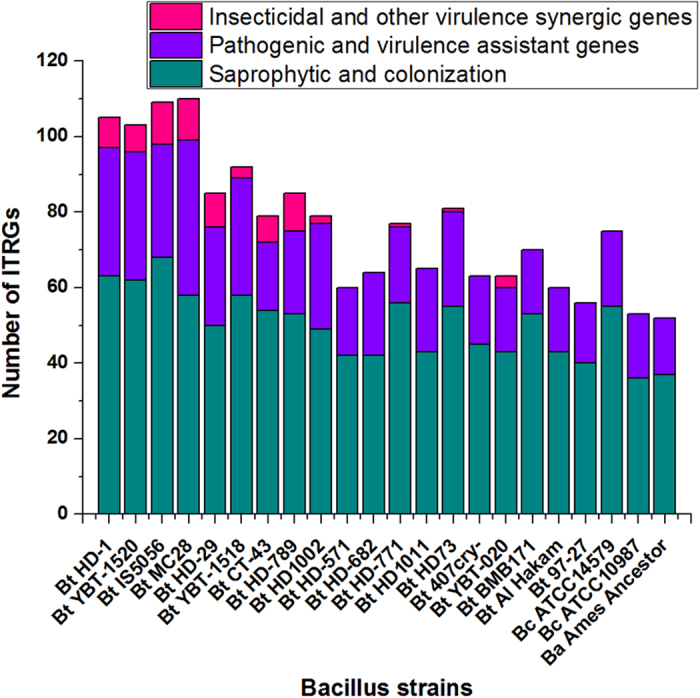
The number of ITRGs in the *B. cereus* group genomes. A gene operon or cluster was counted as one gene.

*B. thuringiensis* encodes a large number of pathogenic factors in its genome and is an insecticidal bacterium that has been used as a biopesticide for many years. Using the reported virulence-associated genes^8^,^22^,^23^ and our unpublished data, a whole-genome InterproScan analysis of strain HD-1 and strain YBT-1520 were used to identify and divide the insecticidal toxicity-related genes and gene clusters (ITRGs) into three groups: insecticidal and other virulence synergic genes, pathogenic and virulence assistant genes, and genes for saprophytic colonization; these genes including 33 families and approximately 100 ITRGs (Supplementary Table S3). Significantly, the results indicated that *Btk* HD-1 and YBT-1520 have 20.0%–87.5% more genes and a 22.2% higher variety of ITRGs than generally, weakly toxic *B. thuringiensis* and other *B. cereus* group bacteria (an operon or a gene cluster counted as one gene, Fig. 3, Supplementary Table S3).

The parasporal crystal protein is the primary insecticidal toxin of *B. thuringiensis*^8^. Strain HD-1 encodes six crystal protein genes, including *cry1Aa*, *cry1Ab*, *cry1Ac*, *cry1Ia*, *cry2Aa,* and *cry2Ab*. Four of these proteins (*cry1Aa*, *cry1Ia*, *cry2Aa,* and *cry2Ab*) are located on the large plasmid pBMB299 with the vegetative insecticidal protein gene *vip3Aa* where they form a pathogenicity island (PAI); two additional proteins are located on plasmids pBMB95 (*cry1Ac*) and pBMB65 (*cry1Ab*). Strain YBT-1520 has nearly identical *cry* genes with strain HD-1 with the exception of *cry1Ab*.

Zwittermycin A (ZmA) is a very important virulence synergic factor that has a broad spectrum of antimicrobial activity and the ability to enhance the insecticidal activity of the Cry protein^24^,^25^. Interestingly, we found a highly conserved bacteriocin PAI in both large plasmids (pBMB431/pBMB422) of strain HD-1 and strain YBT-1520. This PAI includes a ZmA biosynthetic gene cluster^26^,^27^ and two bacteriocin biosynthetic gene clusters (Thurin and Thuricin). And the PAI is flanked by insertion sequences (IS elements). Thurin is a putative novel bacteriocin of unknown function; the gene cluster includes a regulator gene (*thrR*), a precursor gene (*thrA*), a modifier gene (*thrM*), and a transporter gene (*thrT*). The Thuricin gene cluster encodes ten biosynthetic-related genes (Supplementary Table S3) and shows 99% identity with Thuricin H and 17^28^,^29^. These two bacteriocins were divided into saprophytic and colonization groups based on their potential antibacterial activity.

The genes encoding hemolysin and non-hemolysin enterotoxin represent the major important factor involved in the pathogenic and virulence assistant genes group. Although they are conserved among the *B. cereus* group bacteria, *B. thuringiensis* was proven to be safe for use in biological pest control^30^. A comparative genomic analysis revealed that strain HD-1 and strain YBT-1520 possessed a novel copy of the hemolysin BL (HBL) gene operon, which is located in the insecticidal PAI described above (*hblIII1*, Supplementary Table S3). Notably, these two *Btk* also encode five novel proteins belonging to the hemolysin XhlA family, which is a key factor for the full virulence of the insect pathogen *Xenorhabdus nematophila*^31^ (Supplementary Table S3). Additionally, two novel pore-forming toxin genes are located in the large plasmids (*pft* and *nep1*, Supplementary Table S3). The Pft protein showed 33% identity with 213 amino acids (aa) of the parasporin-2 from *B. thuringiensis*^32^ and 38% identity with 222 aa of the hydralysin from *Hydra vulgaris*^33^. This finding suggested that these two genes may play a very important role in pathogenicity. *B. thuringiensis* also encodes a large number of pathogenic assistant factors in the genome, such as phospholipase C (*plc*), sphingomyelinase C (*spl*), and immune inhibitor A (*inhA*)^23^,^34^ (Supplementary Table S3).

The comparative genomic analysis indicated that in addition to the conserved factors among the *B. cereus* group, highly toxic *B. thuringiensis* strains encode more genes related to degradation, adhesion, and recognition. These genes could aid in the effective utilization of insect tissues during the saprophytic colonization stage, especially the enhancins (*bel*), collagenase (*colA*), pectate lyase (*pel*), cell wall hydrolases (*cwl*), extracellular metalloprotease (*mpr*), and cell enveloped S-layer anchor proteases (P < 0.05, T-test, Fig. 3, Supplementary Table S3). These enzymes could potentially introduce competitive advantages to the highly toxicity strains, thereby enabling them to avoid the host immune system and degrade and utilize insect tissues.

### The extra nitrogen metabolism system could make highly toxic B. thuringiensis strains better adapted for survival in the host

**Figure 4:**
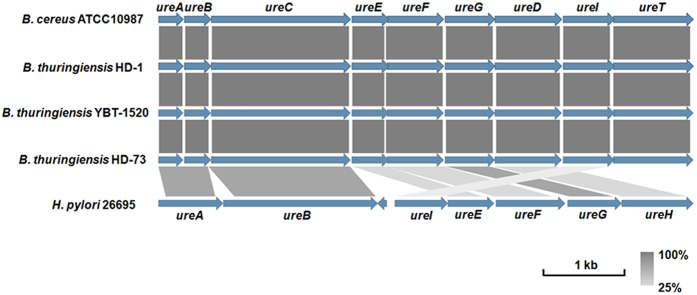
Comparative analysis of the urease gene cluster. Coding sequences (CDSs) are represented by blue arrows. Highly conserved segments of the genes are paired using shaded regions, with the darker shading reflecting greater amino acid identity (from 25% to 100%). The outer scale is given in kilobases.

Most of the *B. cereus* group bacteria only possess the nitrate-nitrite reduction gene cluster for nitrogen source utilization. Previous works have demonstrated that some *B. cereus* group strains lack the nitrate-nitrite reduction gene cluster but use a nine-gene urease synthesis cluster instead in a manner similar to that of *B. cereus* ATCC10987^35^. This study utilized the NCBI database and determined that, in addition to the conserved genes involved in the nitrate-nitrite reduction, all of the *Btk* genomes encoded an extra system for nitrogen source utilization; this system was composed of a nine-gene urease synthesis cluster (*ureA*, *ureB*, *ureC*, *ureE*, *ureF*, *ureG*, *ureD*, *ureI,* and *ureT*, BTK_19245-BTK_19285/YBT1520_19325-YBT1520_19365, Fig. 4). The amino acids had up to 94% identity compared with a specific *B. cereus* strain ATCC10987 isolated from cheese spoilage that only contains the urease synthesis gene cluster but not the nitrate-nitrite reduction system^36^. The urease synthesis cluster in the *Btk* genomes also had homology with some pathogens, including *Helicobacter pylori* (Fig. 4), that use urease for acid acclimation during gastric colonization and acid exposure^37^. There were no insert sequences or transposons flanking the gene cluster, indicating that this extra nitrogen source metabolism system is not a result of horizontal gene transfer but is a specific feature of *Btk*.

### Comparative transcriptomic analysis

To further understand the *B. thuringiensis*-insect interaction, a microarray strategy was used to identify the transcription modulation of the ITRGs during the different growth phases of *Btk* YBT-1520 under culture. Of the 6,668 genes in strain YBT-1520 examined by microarray analysis, 4,998, 4,471, 4,650, and 3,484 genes (75.0%, 67.1%, 69.7% and 52.2%) were transcribed in the four growth phases, respectively. It showed that the transcriptional activity decreased with time, but still a half of the genes in YBT-1520 transcribed in the late stationary growth phase. Additionally, *B. thuringiensis* serovar *chinensis* strain CT-43 was used for comparative transcriptomic analysis in this work. This strain has a similar Cry protein composition to that of *Btk* (especially the same insecticidal PAI). It is also a similar strain to the serovar *thuringiensis*, which was used in early products with less potency than *Btk*. Using the *gatB* (YBT1520_22885) and *rpoA* (YBT1520_00670) genes as reference genes^38^,^39^, the transcriptomic data from the strain YBT-1520 microarray and RNA sequencing from strain CT-43^40^ were normalized for comparison.

### The transcription of ITRGs in B. thuringiensis YBT-1520: genes in the same family are transcribed during different phases

A total of 37.4% of the ITRGs reached the highest transcriptional level in the middle logarithmic growth phase (ML); these ITRGs included *cry1Ia*, 12 of 21 hemolysin family genes, the phospholipase family genes *plc* and *spl*, alveolysin (*alo*), *bel1*, four of seven neutral proteinase genes, a subtilase gene, three metallo-protease genes, four of six collagenase genes, and 12 of 20 S-layer anchor protease genes (Supplementary Fig. S1A, S1B and S1C). The hemolysin family genes were nearly always transcribed at a high level during the log growth phase^34^; interestingly, the novel *hblIII1* operon in the insecticidal PAI had a higher transcription level than the other hemolysin family genes (P < 0.05, T-test, Supplementary Fig. S1B). Additionally, the immune inhibitor A gene *inhA1* was transcribed at a very high level that was close to the transcribed level of the *cry* genes during the forespore formatting phase (P < 0.05, T-test, Supplementary Fig. S1B). Bacterial growth changed from logarithmic growth to stationary growth (early stationary growth phase, ES); a total of 31.3% of the ITRGs reached their highest transcriptional levels during this phase. Notably, the bacteriocin gene clusters (genes encoding Vip3Aa, camelysin, four neutral proteinases, chitinase, four different S-layer anchor proteins, and the pathogenic-related QS regulators PlcR and NprR) showed extra transcriptional activity during this phase (Supplementary Fig. S1A, S1B and S1C). This indicated that during the logarithmic growth phase, *B. thuringiensis* could express various factors (especially the hemolysin BL compomers) to attack the host while also expressing many cofactors (primarily immune inhibitor A and collagenases) to escape host immunity and degrade tissue barriers, as well as antimicrobial peptides to inhibit other competitive bacteria and fungi.

During the middle and late stationary growth phases (MS and LS), the main transcriptional signals of the ITRGs were the Cry protein genes (*cry1Aa*, *cry1Ac*, *cry2Aa,* and *cry2Ab*, P < 0.05, T-test). Interestingly, some pathogenic factors were also expressed, such as hemolysin III1, XhlA3, XhlA4, four subtilases, pectase, metalloproteases, and some S-layer anchor proteases (Supplementary Fig. S1B and S1C). The high levels of expression of the larger number of ITRGs in *Btk* suggested that the bacteria have additional strategies for infection, immune evasion, and cadaver utilization.

We also found that ITRGs in the same protein families were not transcribed during the same phases. The transcription of three immune inhibitor A genes (*inhA*) serves as an example: *inhA1* was up-regulated during the ML phase (5.2-fold compared with the LS phase), but *inhA2* and *inhA3* were up-regulated during the ES phase (2.8-fold compared with the MS and 1.9-fold compared with the LS phases, respectively, Supplementary Fig. S1B and Table S4). Similarly, two enhancin genes (*bel*) were also up-regulated during different phases: *bel1* was up-regulated during the ML phase, whereas *bel2* was up-regulated during the ES phase (6.3- and 3.2-fold, respectively, compared with the LS phase, Supplementary Fig. S1C and Table S4). Most of the saprophytic colonization-related genes were also up-regulated more than three- during the log growth phase (ML and ES), especially the genes from the degradation enzyme families (Supplementary Fig. S1C). The 20 cell envelope S-layer anchor genes could be divided into four families. The three internalin protein genes were regulated at different phases: *ilsa1* was increased in the ML phase (6.1-fold compared with the LS phase), *ilsa2* was increased in the MS phases (52.0-fold compared with the ES phase), and *ilsa3* was increased in the ES phase (1.8-fold compared with the ML phase, Supplementary Fig. S1C and Table S4). The transcriptional pattern of the eight peptidoglycan hydrolase-related genes also differed. Six of these genes were increased in the ML phase (12.5-, 11.3-, 12.0-, 3.4-, 297.4-, and 6.1-fold compared with the ES phase), whereas the remaining two were up-regulated in the LS phase (3.4- and 1.9-fold compared with the ML phase). The other S-layer anchor genes were primarily up-regulated during the ML and ES phases. The different transcriptional phases of the genes in the same protein family suggested that they play a similar role during different infection steps.

**Figure 5:**
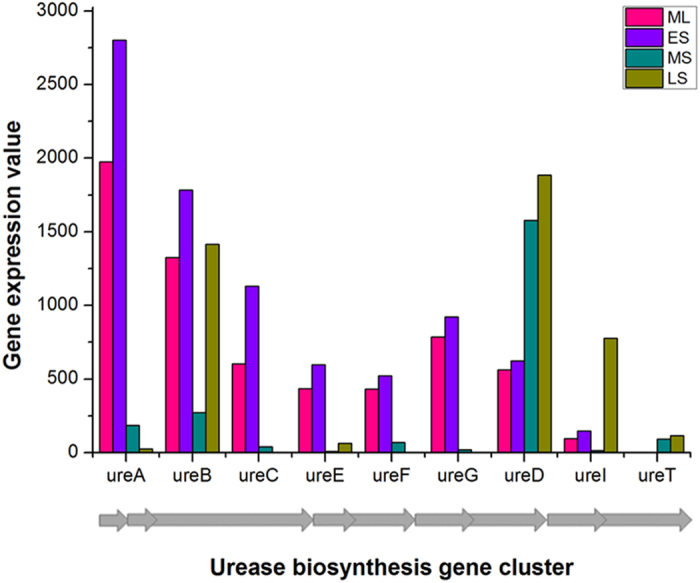
Transcriptomic result of the urease gene cluster in *B. thuringiensis* YBT-1520. The gray arrows represent the corresponding ORFs in the gene cluster. ML: mid log growth phase, ES: early stationary phase, MS: mid stationary phase, LS: late stationary phase.

In addition, the microarray data analysis showed that all 26 novel unique genes around the 740 kb chromosome inversion ends were actively transcribed, including *hblIII2*, *pel*2, *xhlA3*, a regulator gene *pagR*, a reverse transcriptase and a hypothetical protein gene (Supplementary Table S4). And remarkably, the main urease synthesis-related genes were not silenced as previously reported in *B. cereus*^35^ but were actively transcribed during the ES phase even though the transporter genes were up-regulated during the LS phases (Fig. 5, Supplementary Table S4).

### The ITRGs are transcribed more actively and for longer periods of time in the highly toxic strains

**Figure 6:**
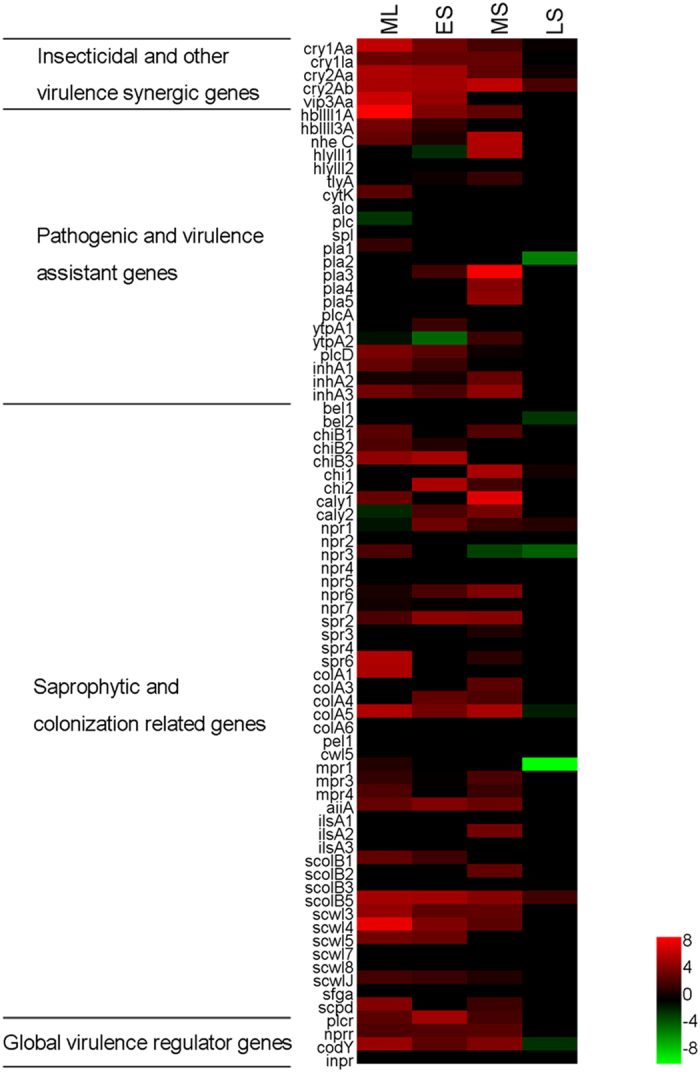
The relative gene expression ratio of conserved ITRGs between *B. thuringiensis* strain YBT-1520 and strain CT-43. The heat map represents the Log2 (fold change) of the expression level of the three groups of conserved ITRGs and the global virulence regulator genes between strain YBT-1520 and strain CT-43. ML, ES, MS, and LS are defined in Fig. 5.

A comparative transcriptomic analysis showed that 8.2% of ITRGs were not transcribed during any phases; 47.6% were not transcribed during the spore formation phase (LS) in strain YBT-1520. In strain CT-43, these values were 19.0% and 79.8%, respectively (Supplementary Fig. S2). The primary transcribed genes in strain CT-43 included *cry1B*, *cry1Aa* and *cry2Aa*; strain YBT-1520 also expressed *cry1Ia*, *cry2Ab,* and *vip3Aa* in addition to the highly transcribed *cry1Aa*, *cry1Ac,* and *cry2Aa* genes, which may provide diversity and a wider spectrum of insect toxicity (Supplementary Table S4). The *cry* and *vip3Aa* genes in strain YBT-1520 showed significantly more activity than the genes in strain CT-43 during the growth phases; the relative expressions of the former were more than tenfold those of the latter (P < 0.05, T-test, Fig. 6). Specifically, the ratio of the *cry2Ab* gene in strain YBT-1520 was increased by more than six-fold at all times (LS, P < 0.05, T-test, Fig. 6). Interestingly, one prevalent explanation for the high toxicity and broad host range spectrum phenotype of *B. thuringiensis* is that there are striking differences in the expression of the conserved *cry* genes between various strains.

Many ITRGs in the pathogenic- and virulence-assisted gene group were conserved in the *B. cereus* group and were highly transcribed in strain YBT-1520 but sparsely transcribed in strain CT-43. These ITRGs included most of the hemolysin family genes, *inhA* and virulence factor *alveolysin* (Fig. 6, Supplementary Table S4). By contrast, nearly all of the protease genes in the saprophytic colonization group in strain YBT-1520 (especially the S-layer anchor-related genes) showed a more than twofold increase in transcription during the three phases compared with strain CT-43 (P < 0.05, T-test, Fig. 6). Specifically, the important pathogenic protease gene camelysin, which can significantly enhance the hemolytic activity of Cyt proteins^41^, was primarily transcribed during the ES phase (Supplementary Table S4) in strain YBT-1520, and the corresponding relative expression (YBT-1520 vs CT-43) in the ES phase was increased two-fold (P < 0.05, T-test, Fig. 6).

Moreover, the ITRGs in the saprophytic colonization group were transcribed for a substantially longer period than those in strain CT-43 during the growth phases. A comparative transcriptomic analysis showed that nearly all of the conserved saprophytic colonization-related protease genes (46 genes in total) in strain YBT-1520 were transcribed during all four growth phases (Supplementary Fig. S1C and S2). However, according to the CT-43 RNA-seq data, 25 of these were only transcribed in one or two growth phases, including the 12 genes that did not exhibit ratios in any of the four growth phases. These genes included a variety of protease families, such as chitinase, neutral protease, subtilisin, collagenase, and several S-layer anchor proteases. This result indicated that highly toxic *B. thuringiensis* strains could express more proteases for a longer period compared with general *B. thuringiensis*, thereby enabling more efficient larval body utilization during the infection process of high toxicity strains.

### Comparative transcriptomic analysis of global virulence regulator genes

To explain the substantially higher transcriptional activity and longer continuous transcription of ITRGs, we compared the transcription of the conserved global virulence regulator genes (primarily the quorum-sensing system (QS) regulators, which regulate several microbial processes, i.e., sporulation, virulence, biofilm formation, conjugation, and the production of extracellular enzymes^34^,^42^). The QS systems were substantially more active in strain YBT-1520 than strain CT-43. During the vegetative growth phase (ML to MS), the relative expression of the QS regulator genes *plcR* and *nprR* were more than sevenfold higher (P<0.05, T-test, Fig. 6). This result is consistent with the higher transcriptional activity detected for many ITRGs (such as the hemolysin genes, which were demonstrated to be controlled by these regulators^34^,^42^).

*codY* of strain YBT-1520, an important global transcriptional regulator that primarily represses the sporulation phase genes, maintained a steady transcription level during the log and stationary growth phases (15.9-, 12.1- and 20.5-fold compared with the LS phase, Supplementary Table S4) and suddenly decreased during the LS growth phase. However, *codY* had an increasing trend during the log and stationary growth phases in strain CT-43 (2.0-, 5.3-, and 18.0-fold compared with the LS phase, P < 0.05, T-test). These results implied that *Btk* has a different regulatory mechanism for development from other *B. thuringiensis* strains.

A global virulence regulator *mga*-like gene (*inpR*, YBT1520_32436) was found upstream of the *vip3Aa* and *cry* genes in the insecticidal PAI. The protein InpR has two Mga helix-turn-helix DNA-binding domains. A BLASTP analysis showed that the 471 aa InpR protein had 25% identity with 213 aa of Mga, which is one of the key global regulators of virulence in Group A *Streptococcus*^43^. InpR also showed 20% identity with 469 aa of the anthrax toxin expression trans-acting positive regulator AtxA from *B. anthracis* plasmid pXO1 and 26% identity with 151 aa of the capsule synthesis trans-acting positive regulator AcpA from pXO2. A phylogenetic analysis indicated that InpR is closely related to the regulators AtxA and AcpA (Supplementary Fig. S3). Moreover, the analysis suggested that the genes in the insecticidal PAI may have their own transcriptional controls. Further analysis showed that the *inpR* in strain YBT-1520 was rarely transcribed during the growth phases; however, in CT-43, *inpR* was transcribed during all four phases. Furthermore, the transcriptional trend was on the rise prior to the release of the spores and parasporal crystals from the mother cell (15.6-, 28.5- and 55.2-fold compared with the LS phase, P < 0.05, T-test). Interestingly, as mentioned above, the relative expression of the important toxins downstream of the *inpR* gene in the PAI, such as *cry1Ia* and *vip3Aa*, were all up-regulated. These results provided an important clue for further study on the function of InpR.

### Functional confirmation of microarray data with qRT-PCR

**Figure 7:**
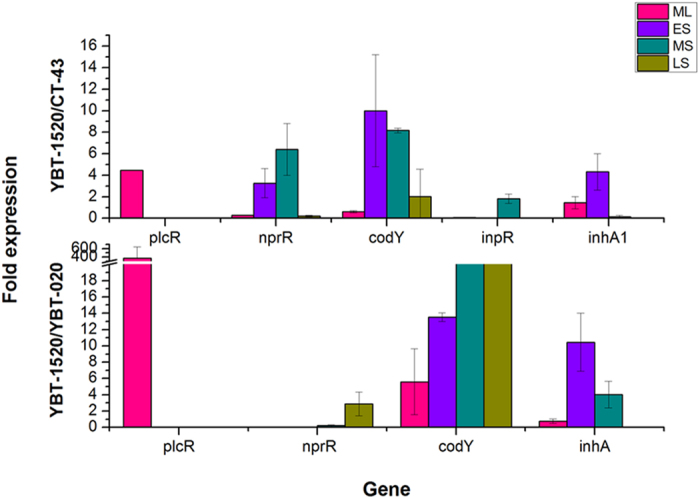
The qRT-PCR analysis of the virulence-related regulators and *inhA* gene in *B. thuringiensis* strains YBT-1520, CT-43 and YBT-020. ML, ES, MS, and LS were defined in Fig. 5. (**A**) The relative gene expression between strain YBT-1520 and strain CT-43. (**B**) The relative gene expression between strain YBT-1520 and strain YBT-020.

Quantitative real-time RT-PCR (qRT-PCR) was performed as a complementary approach to verify the results of the comparative transcriptomic analysis. The qRT-PCR assessments were conducted on RNA samples obtained from the four growth phases of strains YBT-1520, HD-1, CT-43, and YBT-020 under the same culture conditions using primers for *plcR*, *nprR*, *codY*, *inpR,* and *inhA1*. Strain HD-1 exhibited an up-regulated trend that was similar to that of strain YBT-1520. The relative expression of *plcR* from strain YBT-1520 to strain CT-43 was found to be 4.5-fold in the ML phase, and the up-regulation was 370.7-fold compared with strain YBT-020 (P < 0.05, T-test, Fig. 7A,B). The relative expression of *nprR* was up-regulated 2.3- and 4.7-fold during the ES and MS phases, respectively (strain YBT-1520/CT-43, P < 0.05, T-test). The relative expression of *codY* was up-regulated 10.0- and 5.2-fold in the ES and MS phases, respectively (strain YBT-1520/CT-43); compared with strain YBT-020, *codY* was up-regulated 5.6- and 13.5-fold in the ML and ES phases, respectively (P < 0.05, T-test). The relative expression of *inpR* was down-regulated 0.1- and 0.4-fold in the ML and LS phases, respectively, and remained at 1.8-fold during the MS phase (strain YBT-1520/CT-43, P < 0.05, T-test). The relative expression of the conserved *inhA1* gene was increased 1.5-, 4.3-, and 1.7-fold during the ML to MS phases (strain YBT-1520/CT-43); compared with YBT-020, *inhA1* was up-regulated 10.5- and 4.0-fold during the ES and MS phases, respectively (P < 0.05, T-test). These qRT-PCR results were consistent with the comparative transcriptomic results described above. They also confirmed that the transcriptional levels of the global regulator and conserved pathogenic genes in *B. thuringiensis* strains were significantly different under culture conditions.

## Discussion

*B. thuringiensis* usually shows substantial variations in insecticidal toxicity. Thus, more efficient insecticidal strains are used for biopesticide production and in studies of host-pathogen interactions. Remarkably, these strains always contain a large number of plasmids, which makes it difficult to complete genome sequencing using a single high-throughput sequencing strategy. In this study, a multiple sequencing strategy was chosen: after obtaining data via high-throughput technologies, Optical Mapping^44^ was used for chromosome assembly to correct and further assemble the scaffold. Because the various plasmids have no appropriate reference sequence, a genomic BAC library was constructed to determine the relationship between the scaffold and the short contigs. Additionally, a manual analysis of the read data also assisted in closing the gaps. The results confirmed that the “multiple” strategy was effective and practical for complete genome sequencing of complicated bacteria.

Interestingly, comparative genomic analyses indicated that highly insecticidal strains have larger genome sizes and more complicated genome constitutions. This finding is significantly different from that for other pathogens in which the pathogenic strains usually have a smaller genome size than the nonpathogenic strains^45^,^46^. Our results implied that the genome size of *B. thuringiensis* increases as genome complexity increases due to mechanisms involving plasmid content and chromosomal inversion. And we also found a lot of novel ITRGs and other unique genes in these plasmids and the inversion region. This provides the ability of *B. thuringiensis* to adapt to various insect and nematode hosts.

The infection process of *B. thuringiensis* generally involves Cry proteins. These proteins destroy the epithelial cells in the midgut, and the spores germinate and proliferate using the degraded products from the insect cadaver^7^,^8^. Another example includes *Btk* spores (which are not toxic to nematodes alone), which can cause accelerated killing when fed to *C. elegans* with the pore-forming protein Cry5B^47^. These results suggested that *B. thuringiensis* is an insect pathogen but not a toxin-producing soil bacterium. In this study, the insecticidal toxicity-related genes (ITRGs) were summarized and categorized into three groups corresponding to the steps of infection and necrotrophism to investigate the pathogenic mechanisms during infection and the necrotrophic process. Based on the results of the comparative genomic analysis, a hypothesis was proposed to explain the efficient pathogenicity phenotype: 1) The large number of ITRGs could help *B. thuringiensis* effectively kill and utilize nutrient substances from the host; 2) A complicated genome that includes chromosomal inversion rearrangement and a larger number of mobile elements (plasmids, insertion sequences, and introns) not only harbor novel ITRGs but also may provide the evolutionary potential to adapt to a broad spectrum of host environments (insect and nematode) in a manner similar to that of other pathogens; and 3) An active extra nitrogen utilization system may help the bacteria survive and adapt in the host.

Based on this hypothesis, another equally important issue was whether the ITRGs were transcribed or silenced. The microarray analysis showed that the ITRGs were all transcribed to various degrees during the four growth phases. A total of 47.6% and 25.2% of the ITRGs exhibited maximal transcriptional values during the ML and ES growth phases, respectively. In the middle and late stationary growth phases (MS and LS), the main transcriptional signals were the *cry* genes and several pathogenic factors. This finding illustrated the most effective period for the expression of different virulence and pathogenicity-related genes (especially protease genes in the same family), which could be used to improve the model of the *B. thuringiensis* infection process.

Furthermore, this work revealed that ITRGs and conserved global virulence regulator genes in the highly toxic strains were more transcriptionally active than those in the general strain, especially the hemolysins, immune inhibitor A, chitinase, and QS regulators PlcR and NprR. Hence, a fourth explanation should be added to our hypothesis: high expressions of the ITRGs, including ITRGs unique to highly toxic strains and conserved ITRGs among the *B. cereus* group, accelerate the infection process and prevent possible host resistance. Moreover, because the global regulators do not control all of the ITRGs, the high expression of these ITRGs may be under the control of an unknown regulation mechanism. Thus, the different expression levels of ITRGs in these strains may represent an unknown strategy used by *B. thuringiensis* to adapt to various hosts.

## Materials and Methods

### Bacterial strains and culture conditions

*B. thuringiensis* serovar *kurstaki* strain NRRL HD-1 (serotypes H3a, 3b and 3c) was obtained from the U.S. Department of Agriculture’s Agricultural Research Service (ARS) in Peoria, IL. The bacterium was compared with strain 4D1 (BGSC code for HD-1) from the Bacillus Genetic Stock Center (BGSC) in Columbus, USA, and the similar *Btk* strain YBT-1520 was isolated from soil by our laboratory in China. This strain has been used to produce the Mianfeng biopesticide in China^48^ because it showed higher toxicity to the larvae of lepidopteran pests (*Helicoverpa armigera* and *Plutella xylostell*a) compared with the technical standardization strain HD-1 or other *B. thuringiensis* biopesticide strains. All of the *Escherichia coli* and *B. thuringiensis* strains were maintained on Luria-Bertani (LB) agar plates (1% tryptone, 0.5% yeast extract, 0.5% NaCl, and 1.5% agar) supplemented with appropriate antibiotics at 37 and 28 °C, respectively.

### Whole genome sequencing and assembly

Total genomic DNA of *B. thuringiensis* strains HD-1 and YBT-1520 was extracted from the cells according to the procedure of Andrup *et al.*^49^. Due to the complexity of the *B. thuringiensis* genome sequence (especially the abundant and varied plasmids contents), a multiple-step strategy was used for HD-1 whole genome sequencing: 1) After performing a series of strict filtering steps to remove artificial duplication, adapter contamination, and low-quality reads, we obtained 65,899,485 Paired-End (PE) clean reads from different length libraries using the Ilumina Genome Analyzer System (GAIIx) at BGI-Shenzhen, China, representing 1300-fold coverage; 2) a total of 83,060 PE clean reads (14-fold coverage) were obtained using the Roche 454 Genome Sequencer FLX platform (454 GS-FLX) at the Roy J. Carver Biotechnology Center, University of Illinois at Urbana-Champaign, Urbana, IL, USA. A total of 46 scaffolds (>500 bp) of strain HD-1 were obtained using the assembly software SOAP^50^: the N50 length was 1,861,364 bp, the N90 length was 461,121 bp, the maximum length was 2,497,856 bp, and the minimum length was 242,258 bp. The genome alignment generated using the software MAUVE^51^ showed that 15 of the reads overlapped the entire chromosome, whereas the rest may belong to natural plasmids or prophage fragments. Following the analysis of the 454 GS-FLX reads and PCR verifications, we obtained five large scaffolds that overlapped the chromosome of strain HD-1. All reads were mapped onto the contigs for scaffold building by utilizing the paired-end information. After Optical Mapping (OM)^52^ of the HD-1 chromosome was performed at BGI-Shenzhen, the number of scaffolds was reduced to three, and some errors were redressed. The HD-1 chromosome was finished by closing the remaining gaps with PCR. At this point, 21 scaffolds belonged to strain HD-1 plasmids, including some shorter than 500 bp. Thus, we used a whole genome BAC library of strain HD-1 with an approximately 50-kb insert DNA length for complete plasmid assembly. The construction process of the BAC library followed our previous works on *B. thuringiensis* strains YBT-1765 and CT-43^53^,^54^.

For strain YBT-1520, we obtained 8,403,791 clean reads (200-fold coverage) via GAIIx and 86,428 clean reads (14-fold coverage) via 454 GS-FLX. Using strain HD-1 as a reference, the initial assembly produced 1 scaffold that overlapped the chromosome and 15 scaffolds that belonged to plasmids. Following PCR verification, the entire genome of strain YBT-1520 was completed. We obtained a draft genome of strain YBT-1520 using a Sanger Shotgun sequencing strategy in a previous study; this enabled us to complete most of the plasmids^55^,^56^,^57^. The complete genome of strain YBT-1520 in this work was essentially in agreement with the previous draft genome.

### Genome annotation and comparative genomics analysis

The genome annotation was performed by the NCBI Prokaryotic Genomes Automatic Annotation Pipeline Group (http://www.ncbi.nlm.nih.gov/genomes/static/Pipeline.html). The comparative analysis of the chromosomes was performed using BRIG and EasyFig^58^,^59^. A maximum likelihood phylogenomic tree was created using the concatenated amino acid sequences with the program MEGA 5.1^60^.

### Transcriptomic analysis

Harvested bacterial cells were ground in liquid nitrogen with TRIzol and transferred to tubes (10^9^ cells/ml TRIzol) for shaking. RNA isolation and purification were performed according to the TRIzol method^61^. For microarray slide preparation, 6,668 probes were designed according to the whole genomic strain YBT-1520 ORFs and 2,057 ORFs of the *B. cereus* group plasmids in the NCBI database; the microarray was synthesized using the Combimatrix CustomArray platform. cDNA synthesis, labeling, and purification were performed by Advanced Throughput Inc, USA. Four time points within 30 hours of growth were chosen to focus on the differential expression of the pathogenic factors: the 6th, 9th, 18th, and 27th hours represent the middle logarithmic growth phase (ML), early stationary growth phase (ES), middle stationary growth phase with forespore formatting (MS), and late stationary growth phase with some spores and parasporal crystals release (LS), respectively (Supplementary Fig. S4). The R platform, Cluster 3.0, and Tree View 1.1.6 were used for filtering, normalization, and cluster analysis. P-values were computed using the false discovery rate correction of 0.05.

### Quantitative real-time PCR

Prior to reverse transcription (RT), total RNA was subjected to PCR using primers specific for 16S rDNA and 23S rDNA to exclude the possibility of genomic DNA contamination. RNA (40 ng) was converted into cDNA using the QuantiTec reverse transcription kit (Qiagen) as recommended by the manufacturer. Quantitative real-time RT-PCRs (qRT-PCRs) were performed using the QuantiFast SYBR green PCR kit (Qiagen) according to the manufacturer’s protocol and were run in a LightCycler instrument (ABI). The entire experiment was repeated two times on RNA samples extracted from the four growth phases of the *B. thuringiensis* strains. Changes in mRNA levels were calculated using the 2^−ΔΔCT^ method^62^.

## Additional Information

**Accession numbers**: The genome sequences of strain HD-1 and strain YBT-1520 have been submitted to the GenBank of NCBI (http://www.ncbi.nlm.nih.gov/) under accession nos CP004870-CP004883 and CP004858-CP004869, respectively. Microarray data for strain YBT-1520 are available in the ArrayExpress database (www.ebi.ac.uk/arrayexpress) under accession number E-MTAB-2162.

**How to cite this article**: Zhu, L. *et al.* Genomic and transcriptomic insights into the efficient entomopathogenicity of *Bacillus thuringiensis*. *Sci. Rep.* **5**, 14129; doi: 10.1038/srep14129 (2015).

## Supplementary Material

Supplementary Information

## Figures and Tables

**Table 1:** Features of *B. thuringiensis* strains and some typical *B. cereus* group genomes.

*Bacillus* strains		Insecticidaltoxicity*	ChromosomeSize (bp)	Totalgenomesize (bp)	AverageG + Ccontent(%)	No. ofplasmids	No. ofGene	No. oftRNAs	No. ofrRNAs	References orBioProject no.
*Bacillus thuringiensis*	HD-1	High	5,631,672	6,767,044	34.92	13	6,928	94, plus 1 on plasmid	41	This study
	YBT-1520		5,602,565	6,580,536	34.91	11	6,720	98, plus 1 on plasmid	39	This study
	IS5056		5,491,935	6,771,593	34.91	14	6,755	85	39	10
	MC28		5,414,461	6,694,500	34.92	7	6,658	73, plus 2 on plasmids	45	12
	HD-29		5,701,188	6,742,233	34.96	10	6,903	114, plus 1 on plasmid	42	63
	YBT-1518		6,002,284	6,672,921	35.29	6	6,877	91, plus 3 on plasmids	44	19
	4AA1		5,652,292	6,179,896	35.09	6	6,337	98	39	PRJNA224116
	CT-43	General	5,486,830	6,151,150	35.12	10	6,270	85	39	14
	HD-789		5,495,278	6,334,630	35.18	6	6,525	104, plus 17 on plasmid	42	64
	HD1002		5,491,311	6,572,702	35.07	7	6,845	116, plus 17 on plasmid	39	PRJNA236049
	HD-571		5,256,240	5,312,179	35.41	1	5,538	104	42	PRJNA238070
	HD-682		5,213,295	5,291,389	35.48	3	5,546	105	42	PRJNA238078
	HD-771		5,886,036	6,438,373	35.04	8	6,591	98, plus 1 on plasmid	36	PRJNA171845
	HD1011		5,232,696	6,093,375	35.15	4	6,305	104	42	PRJNA238081
	HD73	Weak	5,646,799	5,908,575	35.19	7	6,169	104	36	9
	407 cry-		5,500,501	6,134,344	35.02	9	6,455	107, plus 31 on plasmid	42	11
	YBT-020		5,355,490	5,682,383	35.38	2	5,872	105, plus 2 on plasmids	42	15
	BMB171		5,330,088	5,643,051	35.19	1	5,760	102	42	13
	Al Hakam		5,257,091	5,313,030	35.41	1	5,537	104	42	65
	97-27		5,237,682	5,314,794	35.36	1	5,343	105	41	16
*Bacillus cereus*	ATCC14579	–	5,411,809	5,427,083	35.31	1	5,494	108	39	66
	ATCC10987		5,224,283	5,432,652	35.52	1	5,609	97	36	36
*Bacillus anthracis*	Ames Ancestor	–	5,227,419	5,503,926	35.26	2	5,862	95	33	67

*The different insecticidal toxicity was divided based on the reported works and serotypes. And *B. cereus*/*B. anthracis* were marked by “–” for no insecticidal toxicity.
