# Supplementary material for: Genomic and transcriptomic insights into the efficient entomopathogenicity of Bacillus thuringiensis
Source: Sci Rep. 2015 Sep 28;5:14129. doi: 10.1038/srep14129 (PMC4585936; doi:10.1038/srep14129)
Supplement: Supplementary Information [file srep14129-s1.pdf]

Supplementary information:

Genomic and transcriptomic insights into the efficient entomopathogenicity of *Bacillus thuringiensis*

Lei Zhu, Donghai Peng, Yueying Wang, Weixing Ye, Jinshui Zheng, Changming Zhao, Dongmei Han, Ce Geng, Lifang Ruan, Jin He, Ziniu Yu, Ming Sun#

Figures:

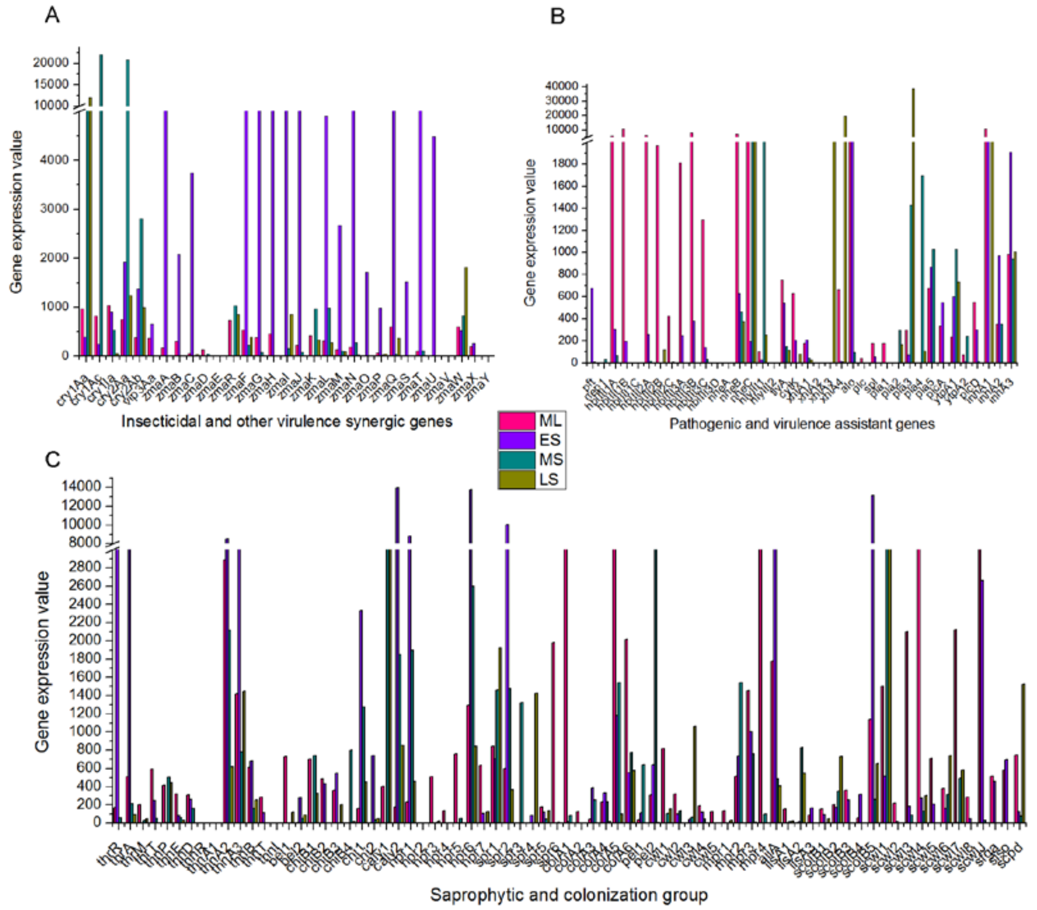

Figure S1 Transcriptomic analysis of the ITRGs in *B. thuringiensis* YBT-1520. A, B and C represent the insecticidal and other virulence synergic genes, pathogenic and virulence assistant genes and saprophytic and colonization group, respectively. ML, ES, MS, and LS are defined in Figure 5.

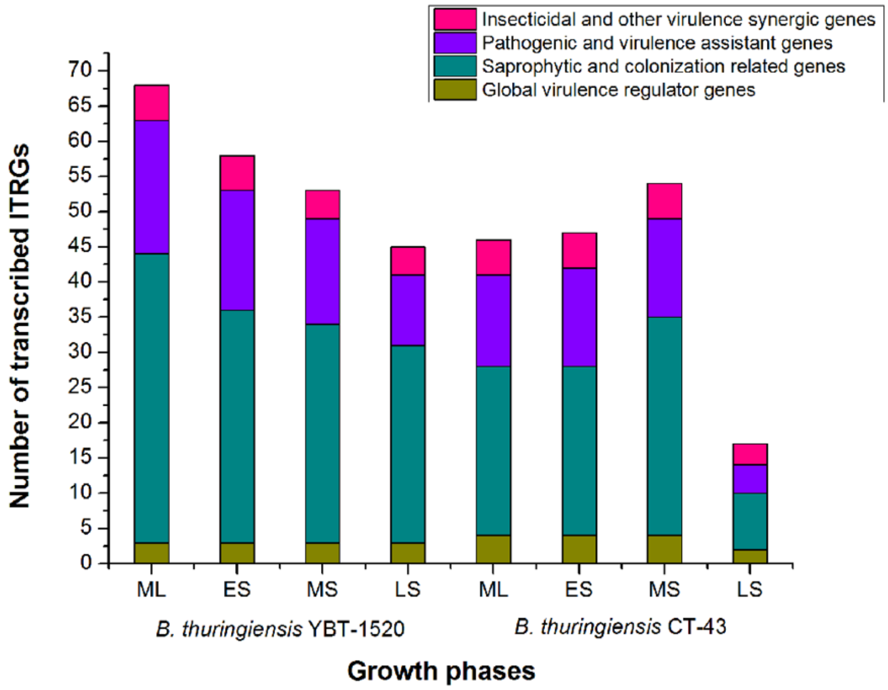

Figure S2 The number of transcribed conserved ITRGs between *B. thuringiensis* strain YBT-1520 and strain CT-43. Pink, insecticidal and other virulence synergic genes; violet, pathogenic and virulence assistant genes; dark cyan, saprophytic and colonization-related genes; and dark yellow, global virulence regulator genes. A gene operon or cluster was counted as one gene. ML, ES, MS, and LS are defined in Figure 5.

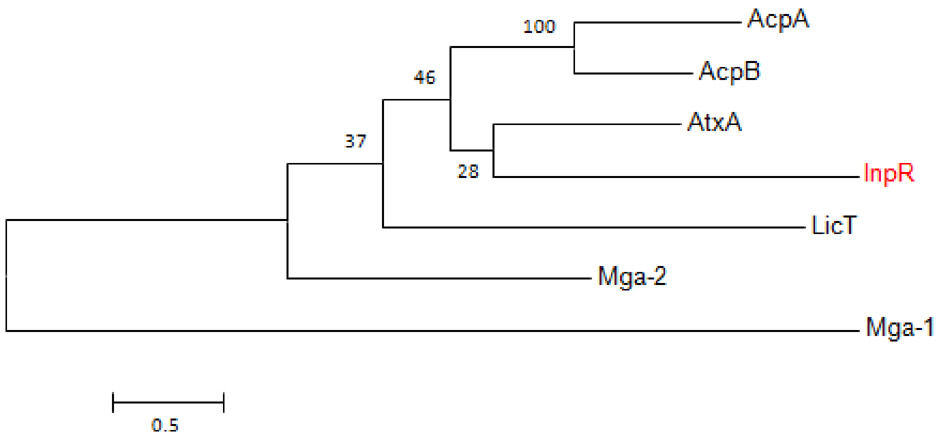

Figure S3 Best maximum likelihood (ML) phylogenetic analysis of the *inpR* gene with homologs from *Bacillus* and *Streptococcus*. The bootstrap values are notated with 500 replicates.

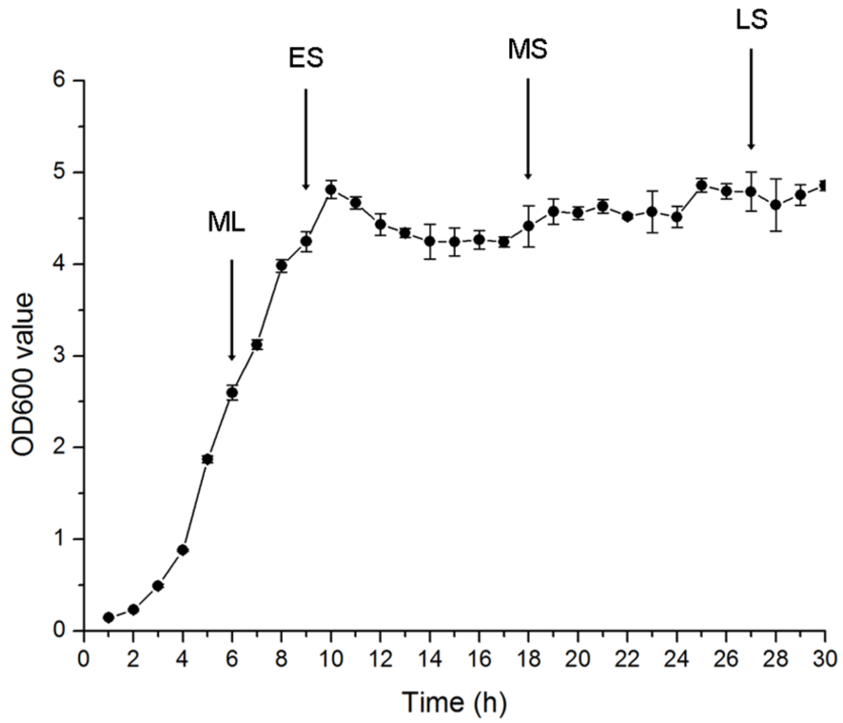

Figure S4 The growth curve of *B. thuringiensis* YBT-1520. The four time points chosen for microarray analysis are marked by arrows. ML, ES, MS, and LS are defined in Figure 5.

Table S1. Plasmid content of *B. thuringiensis* serovar *kurstaki* HD-1 and YBT-1520.

Strain	Plasmid	Size (bp)	G+C content (%)	No. of ORFs	Insecticidal toxin genes
HD-1	pBMB431	431,546	32.6	354	Zwittermycin A biosynthetic gene cluster
	pBMB299	299,843	33.2	275	*cry1Aa* , *cry1Ia* , *cry2Aa* , *cry2Ab* , *vip3Aa*
	pBMB95	95,983	31.5	112	*cry1Ac*
	pBMB74	74,480	33.7	86	-
	pBMB65	65,873	34.8	63	*cry1Ab*
	pBMB64	64,522	31.9	72	-
	pBMB46	46,634	35.4	68	-
	pBMBLin15	14,870	40.1	22	-
	pBMB14	14,721	31	23	-
	pBMB8513	8,513	30.9	10	-
	pBMB8240	8,240	29.7	8	-
	pBMB7635	7,635	32.2	12	-
	pBMB2062	2,062	34.9	3	-
YBT-1520	pBMB422	422,692	32.6	348	Zwittermycin A biosynthetic gene cluster
	pBMB293	293,574	33.2	277	*cry1Aa* , *cry1Ia* , *cry2Aa* , *cry2Ab* , *vip3Aa*
	pBMB94	94,568	31.4	108	*cry1Ac*
	pBMB67	67,159	32.4	79	-
	pBMB53	53,838	34.4	54	-
	pBMB11	11,769	31.8	20	-
	pBMB8513	8,513	30.9	10	-
	pBMB8240	8,240	29.7	8	-
	pBMB7921	7,921	32.3	11	-
	pBMB7635	7,635	32.2	12	-
	pBMB2062	2,062	34.9	3	-

Table S2 *B. thuringiensis* with draft genomes and all sequenced *B. cereus* /*B. anthracis* used in this stud[illegible]

Strain name	Species	Insecticidal toxicity[a]	Genome size (Mb)	No. of plasmids[b]	NCBI BioProject no.
T03a001	*Bacillus thuringiensis*	High	5.52757	-	PRJNA55217
HD600	*Bacillus thuringiensis*	High	6.91681	-	PRJNA224116
Leapi01	*Bacillus thuringiensis*	High	6.15672	-	PRJNA221253
Hu4-2	*Bacillus thuringiensis*	High	6.45863	-	PRJNA221254
ATCC 35646	*Bacillus thuringiensis*	High	5.88084	-	PRJNA54295
BGSC 4Y1	*Bacillus thuringiensis*	General	5.62591	-	PRJNA55219
T01001	*Bacillus thuringiensis*	General	6.32312	-	PRJNA55225
T04001	*Bacillus thuringiensis*	General	6.10775	-	PRJNA55221
T13001	*Bacillus thuringiensis*	General	6.03751	-	PRJNA55227
BGSC 4AJ1	*Bacillus thuringiensis*	General	6.48902	-	PRJNA55215
ATCC 10792	*Bacillus thuringiensis*	General	6.26014	-	PRJNA55229
BGSC 4AW1	*Bacillus thuringiensis*	General	5.48884	-	PRJNA55231
BGSC 4BA1	*Bacillus thuringiensis*	General	6.03148	-	PRJNA55233
BGSC 4BD1	*Bacillus thuringiensis*	General	6.23120	-	PRJNA55235
BGSC 4CC1	*Bacillus thuringiensis*	General	6.00260	-	PRJNA55237
DAR 81934	*Bacillus thuringiensis*	General	5.94380	-	PRJNA198418
DB27	*Bacillus thuringiensis*	General	6.08626	-	PRJNA224116
NBIN-866	*Bacillus thuringiensis*	General	5.81846	-	PRJNA224116
T01-328	*Bacillus thuringiensis*	General	7.08969	-	PRJNA201353
LM1212	*Bacillus thuringiensis*	General	6.06218	-	PRJNA224116
JM-Mgvxx-63	*Bacillus thuringiensis*	General	4.93180	-	PRJNA224116
NA205-3	*Bacillus thuringiensis*	General	6.51005	-	PRJNA224116
XL6	*Bacillus thuringiensis*	General	5.70752	-	PRJNA224116
AK47	*Bacillus thuringiensis*	General	6.36548	-	PRJNA224116
Et10/1	*Bacillus thuringiensis*	General	9.83967	-	PRJNA224116
Lr7/2	*Bacillus thuringiensis*	General	5.61003	-	PRJNA224116
Lr3/2	*Bacillus thuringiensis*	General	5.56608	-	PRJNA224116
IBL 200	*Bacillus thuringiensis*	Weak	6.73179	-	PRJNA55239
IBL 4222	*Bacillus thuringiensis*	Weak	6.61243	-	PRJNA55241
4Q7	*Bacillus thuringiensis*	Weak	5.04044	-	PRJNA224116
ATCC 14579	*Bacillus cereus*	-	5.42708	1	PRJNA57975
AH621	*Bacillus cereus*	-	5.67481	-	PRJNA55161
Rock4-18	*Bacillus cereus*	-	5.92363	-	PRJNA55193
ATCC 10987	*Bacillus cereus*	-	5.43265	1	PRJNA57673
E33L	*Bacillus cereus*	-	5.84323	5	PRJNA58103
Q1	*Bacillus cereus*	-	5.50621	2	PRJNA58529
B4264	*Bacillus cereus*	-	5.41904	0	PRJNA58757
AH187	*Bacillus cereus*	-	5.59986	4	PRJNA58753
G9842	*Bacillus cereus*	-	5.73682	2	PRJNA58759
AH820	*Bacillus cereus*	-	5.58883	3	PRJNA58751
03BB102	*Bacillus cereus*	-	5.44931	1	PRJNA59299
CI	*Bacillus cereus*	-	5.48665	3	PRJNA50615
F837/76	*Bacillus cereus*	-	5.28850	2	PRJNA83611

NC7401	*Bacillus cereus*	-	5.55203	5	PRJNA82815
FRI-35	*Bacillus cereus*	-	5.38232	4	PRJNA173403
FT9	*Bacillus cereus*	-	5.22366	0	PRJNA224116
03BB87	*Bacillus cereus*	-	5.71894	2	PRJNA224116
D17	*Bacillus cereus*	-	5.59036	1	PRJNA224116
FM1	*Bacillus cereus*	-	5.69776	1	PRJNA224116
3a	*Bacillus cereus*	-	5.64230	3	PRJNA238242
G9241	*Bacillus cereus*	-	5.72007	3	PRJNA224116
ATCC 4342	*Bacillus cereus*	-	5.30630	1	PRJNA224116
03BB108	*Bacillus cereus*	-	6.06873	7	PRJNA224116
Al Hakam	*Bacillus cereus*	-	5.67696	6	PRJNA224116
S2-8	*Bacillus cereus*	-	5.64247	3	PRJNA224116
m1293	*Bacillus cereus*	-	5.26973	-	PRJNA55163
AH1273	*Bacillus cereus*	-	5.79050	-	PRJNA55205
ATCC 10876	*Bacillus cereus*	-	5.93902	-	PRJNA55177
BGSC 6E1	*Bacillus cereus*	-	5.73018	-	PRJNA55157
172560W	*Bacillus cereus*	-	5.69954	-	PRJNA55151
MM3	*Bacillus cereus*	-	5.54706	-	PRJNA55159
R309803	*Bacillus cereus*	-	5.58625	-	PRJNA55153
m1550	*Bacillus cereus*	-	5.24649	-	PRJNA55155
BDRD-ST24	*Bacillus cereus*	-	5.43617	-	PRJNA55165
BDRD-ST26	*Bacillus cereus*	-	5.56803	-	PRJNA55167
BDRD-ST196	*Bacillus cereus*	-	5.57657	-	PRJNA55169
BDRD-Cer4	*Bacillus cereus*	-	5.39745	-	PRJNA55173
95/8201	*Bacillus cereus*	-	5.58406	-	PRJNA55175
Rock1-3	*Bacillus cereus*	-	5.85985	-	PRJNA55179
Rock1-15	*Bacillus cereus*	-	5.76628	-	PRJNA55181
Rock3-28	*Bacillus cereus*	-	6.04150	-	PRJNA55183
Rock3-29	*Bacillus cereus*	-	5.87811	-	PRJNA55185
Rock3-42	*Bacillus cereus*	-	5.20290	-	PRJNA55187
Rock3-44	*Bacillus cereus*	-	4.99980	-	PRJNA55189
Rock4-2	*Bacillus cereus*	-	5.76999	-	PRJNA55191
F65185	*Bacillus cereus*	-	6.13318	-	PRJNA55195
AH603	*Bacillus cereus*	-	5.79945	-	PRJNA55197
AH676	*Bacillus cereus*	-	5.59475	-	PRJNA55199
AH1271	*Bacillus cereus*	-	5.65670	-	PRJNA55201
AH1272	*Bacillus cereus*	-	5.78954	-	PRJNA55203
F	*Bacillus cereus*	-	5.25577	-	PRJNA198416
W	*Bacillus cereus*	-	5.24057	-	PRJNA54483
AH1134	*Bacillus cereus*	-	5.90875	-	PRJNA54485
NVH0597-99	*Bacillus cereus*	-	5.34870	-	PRJNA54831
LCT-BC244	*Bacillus cereus*	-	5.15688	-	PRJNA190362
VDM062	*Bacillus cereus*	-	6.01159	-	PRJNA181707
VDM034	*Bacillus cereus*	-	5.97033	-	PRJNA181706
VDM022	*Bacillus cereus*	-	6.31247	-	PRJNA181705
VD200	*Bacillus cereus*	-	6.27422	-	PRJNA181704
VD169	*Bacillus cereus*	-	5.95137	-	PRJNA181703
VD166	*Bacillus cereus*	-	6.14684	-	PRJNA181702

VD156	*Bacillus cereus*	-	6.42130	-	PRJNA181701
VD154	*Bacillus cereus*	-	6.30559	-	PRJNA181700
VD148	*Bacillus cereus*	-	5.74292	-	PRJNA181699
VD115	*Bacillus cereus*	-	6.43502	-	PRJNA181698
VD107	*Bacillus cereus*	-	5.37228	-	PRJNA181697
VD102	*Bacillus cereus*	-	5.77333	-	PRJNA181696
VD078	*Bacillus cereus*	-	6.10852	-	PRJNA181695
VD048	*Bacillus cereus*	-	6.03857	-	PRJNA181694
IS075	*Bacillus cereus*	-	5.81377	-	PRJNA181653
VD022	*Bacillus cereus*	-	6.68524	-	PRJNA181654
VD142	*Bacillus cereus*	-	5.92391	-	PRJNA181655
AND1407	*Bacillus cereus*	-	5.50502	-	PRJNA181656
BAG1O-2	*Bacillus cereus*	-	5.58534	-	PRJNA181657
BAG1X1-2	*Bacillus cereus*	-	5.85620	-	PRJNA181658
BAG1X1-3	*Bacillus cereus*	-	6.03048	-	PRJNA181659
HuA4-10	*Bacillus cereus*	-	5.80330	-	PRJNA181682
HuB4-10	*Bacillus cereus*	-	5.64213	-	PRJNA181685
HuB5-5	*Bacillus cereus*	-	5.80297	-	PRJNA181686
ISP3191	*Bacillus cereus*	-	5.37112	-	PRJNA181687
MC67	*Bacillus cereus*	-	5.90923	-	PRJNA181688
HuA2-4	*Bacillus cereus*	-	5.72204	-	PRJNA181681
MSX-A1	*Bacillus cereus*	-	6.06670	-	PRJNA181689
VD045	*Bacillus cereus*	-	6.51428	-	PRJNA181693
MSX-A12	*Bacillus cereus*	-	5.77891	-	PRJNA181690
VD014	*Bacillus cereus*	-	6.20418	-	PRJNA181692
MSX-D12	*Bacillus cereus*	-	5.61567	-	PRJNA181691
BAG5X1-1	*Bacillus cereus*	-	5.63517	-	PRJNA181670
CER074	*Bacillus cereus*	-	5.60356	-	PRJNA181678
CER057	*Bacillus cereus*	-	5.62838	-	PRJNA181677
BAG6X1-2	*Bacillus cereus*	-	5.11668	-	PRJNA181675
BAG5X2-1	*Bacillus cereus*	-	5.85529	-	PRJNA181671
BAG5O-1	*Bacillus cereus*	-	5.88479	-	PRJNA181669
BAG4X12-1	*Bacillus cereus*	-	5.82559	-	PRJNA181667
BAG4O-1	*Bacillus cereus*	-	5.72636	-	PRJNA181666
BAG3X2-2	*Bacillus cereus*	-	5.86569	-	PRJNA181665
BAG3X2-1	*Bacillus cereus*	-	5.67960	-	PRJNA181664
BAG2X1-2	*Bacillus cereus*	-	5.58965	-	PRJNA181661
BAG2X1-3	*Bacillus cereus*	-	5.25057	-	PRJNA181662
BAG3O-2	*Bacillus cereus*	-	5.85602	-	PRJNA181663
BAG2X1-1	*Bacillus cereus*	-	5.18894	-	PRJNA181660
BAG4X2-1	*Bacillus cereus*	-	5.72513	-	PRJNA181668
BAG6O-1	*Bacillus cereus*	-	5.89881	-	PRJNA181672
BAG6O-2	*Bacillus cereus*	-	5.74723	-	PRJNA181673
BAG6X1-1	*Bacillus cereus*	-	5.52723	-	PRJNA181674
BtB2-4	*Bacillus cereus*	-	5.66198	-	PRJNA181676
HD73	*Bacillus cereus*	-	5.87919	-	PRJNA181679
HuA2-1	*Bacillus cereus*	-	6.46528	-	PRJNA181680
HuB1-1	*Bacillus cereus*	-	5.74395	-	PRJNA181683

HuB2-9	*Bacillus cereus*	-	5.76967	-	PRJNA181684
TIAC219	*Bacillus cereus*	-	6.82029	-	PRJNA203449
Schrouff	*Bacillus cereus*	-	6.26708	-	PRJNA203460
HuA3-9	*Bacillus cereus*	-	6.24132	-	PRJNA203472
HuA2-9	*Bacillus cereus*	-	5.83756	-	PRJNA203471
BAG1X1-1	*Bacillus cereus*	-	6.54283	-	PRJNA203462
VD133	*Bacillus cereus*	-	6.78691	-	PRJNA203483
VDM019	*Bacillus cereus*	-	6.00844	-	PRJNA203490
BAG1X2-1	*Bacillus cereus*	-	6.53486	-	PRJNA203463
BAG1X2-2	*Bacillus cereus*	-	6.85877	-	PRJNA203464
BAG1X2-3	*Bacillus cereus*	-	6.53174	-	PRJNA203465
VD196	*Bacillus cereus*	-	6.43303	-	PRJNA203488
VD021	*Bacillus cereus*	-	6.09784	-	PRJNA203480
BAG1O-1	*Bacillus cereus*	-	6.03922	-	PRJNA203461
K-5975c	*Bacillus cereus*	-	6.31975	-	PRJNA203478
IS845/00	*Bacillus cereus*	-	5.87634	-	PRJNA203476
IS195	*Bacillus cereus*	-	5.89227	-	PRJNA203475
MC118	*Bacillus cereus*	-	5.60841	-	PRJNA203479
BAG2O-1	*Bacillus cereus*	-	6.69791	-	PRJNA203466
BAG2O-3	*Bacillus cereus*	-	5.81244	-	PRJNA203467
HuB4-4	*Bacillus cereus*	-	6.54416	-	PRJNA203474
BAG5X12-1	*Bacillus cereus*	-	5.72583	-	PRJNA203468
BMG1.7	*Bacillus cereus*	-	6.49649	-	PRJNA203469
HuB13-1	*Bacillus cereus*	-	6.72989	-	PRJNA203473
VD131	*Bacillus cereus*	-	6.11847	-	PRJNA203482
HuA2-3	*Bacillus cereus*	-	5.71703	-	PRJNA203470
VD146	*Bacillus cereus*	-	6.10938	-	PRJNA203486
VD214	*Bacillus cereus*	-	5.69015	-	PRJNA203489
VDM053	*Bacillus cereus*	-	6.11070	-	PRJNA203491
VD136	*Bacillus cereus*	-	5.26609	-	PRJNA203484
ISP2954	*Bacillus cereus*	-	6.74644	-	PRJNA203477
VD118	*Bacillus cereus*	-	5.78295	-	PRJNA203481
VD140	*Bacillus cereus*	-	5.70940	-	PRJNA203485
VD184	*Bacillus cereus*	-	6.33203	-	PRJNA203487
VDM006	*Bacillus cereus*	-	5.42693	-	PRJNA203492
VDM021	*Bacillus cereus*	-	5.47077	-	PRJNA203493
B5-2	*Bacillus cereus*	-	5.84129	-	PRJNA203495
BAG3O-1	*Bacillus cereus*	-	5.87504	-	PRJNA203494
BAG2O-2	*Bacillus cereus*	-	5.89103	-	PRJNA209029
BAG1O-3	*Bacillus cereus*	-	5.85119	-	PRJNA209028
LCT-BC25	*Bacillus cereus*	-	5.15451	-	PRJNA224116
LCT-BC235	*Bacillus cereus*	-	5.14873	-	PRJNA224116
F1-15	*Bacillus cereus*	-	5.60021	-	PRJNA224116
13061	*Bacillus cereus*	-	5.47042	-	PRJNA224116
tsu1	*Bacillus cereus*	-	5.81258	-	PRJNA256220
H3081.97	*Bacillus cereus*	-	5.54331	5	PRJNA54833
SJ1	*Bacillus cereus*	-	5.15640	-	PRJNA50375
A1	*Bacillus cereus*	-	5.66734	-	PRJNA224116

BcFL2013	*Bacillus cereus*	-	5.46244	-	PRJNA224116
MIT0214	*Bacillus cereus*	-	5.59424	-	PRJNA224116
105Ne	*Bacillus cereus*	-	5.55488	-	PRJNA224116
Lr 4/2	*Bacillus cereus*	-	6.22518	-	PRJNA224116
Ames	*Bacillus anthracis*	-	5.22729	0	PRJNA57909
Sterne	*Bacillus anthracis*	-	5.22866	0	PRJNA58091
Ames Ancestor'	*Bacillus anthracis*	-	5.50393	2	PRJNA58083
CDC 684	*Bacillus anthracis*	-	5.50676	2	PRJNA59303
A0248	*Bacillus anthracis*	-	5.50393	2	PRJNA59385
H9401	*Bacillus anthracis*	-	5.49547	2	PRJNA162021
A16R	*Bacillus anthracis*	-	5.40945	1	PRJNA224116
A16	*Bacillus anthracis*	-	5.50450	2	PRJNA224116
SVA11	*Bacillus anthracis*	-	5.48752	2	PRJNA224116
HYU01	*Bacillus anthracis*	-	5.49012	2	PRJNA224116
2000031021	*Bacillus anthracis*	-	5.33174	1	PRJNA224116
Vollum	*Bacillus anthracis*	-	5.50619	2	PRJNA224116
Cvac02	*Bacillus anthracis*	-	5.22717	0	PRJNA224116
Han	*Bacillus anthracis*	-	5.22543	0	PRJNA224116
Ames A0462	*Bacillus anthracis*	-	5.50391	2	PRJNA224116
PAK-1	*Bacillus anthracis*	-	5.40338	1	PRJNA224116
Vollum 1B	*Bacillus anthracis*	-	5.50663	2	PRJNA224116
K3	*Bacillus anthracis*	-	5.50499	2	PRJNA224116
Ohio ACB	*Bacillus anthracis*	-	5.49834	2	PRJNA224116
SK-102	*Bacillus anthracis*	-	5.50566	2	PRJNA224116
Pasteur	*Bacillus anthracis*	-	5.29480	1	PRJNA224116
Sterne	*Bacillus anthracis*	-	5.40912	1	PRJNA224116
BA1015	*Bacillus anthracis*	-	5.49118	2	PRJNA224116
BA1035	*Bacillus anthracis*	-	5.48726	2	PRJNA224116
RA3	*Bacillus anthracis*	-	5.48987	2	PRJNA224116
V770-NP-1R	*Bacillus anthracis*	-	5.41040	1	PRJNA224116
2002013094	*Bacillus anthracis*	-	5.60108	2	PRJNA224116
Ames_BA1004	*Bacillus anthracis*	-	5.50397	2	PRJNA224116
Canadian_bison	*Bacillus anthracis*	-	5.50577	2	PRJNA224116
Turkey32	*Bacillus anthracis*	-	5.50530	2	PRJNA224116
A1144	*Bacillus anthracis*	-	5.47706	2	PRJNA224116
A2012	*Bacillus anthracis*	-	5.37006	2	PRJNA54101
delta Sterne	*Bacillus anthracis*	-	5.22965	0	PRJNA224116
BFV	*Bacillus anthracis*	-	5.50835	2	PRJNA224116
A0157	*Bacillus anthracis*	-	5.32224	1	PRJNA224116
Pollino	*Bacillus anthracis*	-	5.50389	2	PRJNA224116
8903-G	*Bacillus anthracis*	-	5.50477	2	PRJNA224116
9080-G	*Bacillus anthracis*	-	5.50866	2	PRJNA224116
52-G	*Bacillus anthracis*	-	5.50444	2	PRJNA224116
Smith 1013	*Bacillus anthracis*	-	5.28699	1	PRJNA224116
CNEVA-9066	*Bacillus anthracis*	-	5.48868	-	PRJNA54133
A1055	*Bacillus anthracis*	-	5.37062	-	PRJNA54131
Vollum	*Bacillus anthracis*	-	5.48846	-	PRJNA54135
Kruger B	*Bacillus anthracis*	-	5.47001	-	PRJNA54105

Western North A	*Bacillus anthracis*	-	5.51135	-	PRJNA54107
Australia 94	*Bacillus anthracis*	-	5.50456	-	PRJNA54137
Tsiankovskii-I	*Bacillus anthracis*	-	5.50473	-	PRJNA54481
A0488	*Bacillus anthracis*	-	5.39217	-	PRJNA54995
A0193	*Bacillus anthracis*	-	5.39288	-	PRJNA55005
A0442	*Bacillus anthracis*	-	5.37484	-	PRJNA54997
A0465	*Bacillus anthracis*	-	5.40715	-	PRJNA55001
A0174	*Bacillus anthracis*	-	5.29191	-	PRJNA55003
Carbosap	*Bacillus anthracis*	-	5.40767	-	PRJNA224116
Zimbabwe 89	*Bacillus anthracis*	-	5.46092	-	PRJNA224116
A.Br.003	*Bacillus anthracis*	-	5.48772	-	PRJNA224116
A0389	*Bacillus anthracis*	-	5.42040	2	PRJNA54999
Heroin Ba4599	*Bacillus anthracis*	-	5.46838	-	PRJNA190302
UR-1	*Bacillus anthracis*	-	5.44661	-	PRJNA180871
BF1	*Bacillus anthracis*	-	5.43453	1	PRJNA180907
Carbosap	*Bacillus anthracis*	-	5.40297	-	PRJNA198410
3154	*Bacillus anthracis*	-	5.00269	-	PRJNA198411
3166	*Bacillus anthracis*	-	5.50514	-	PRJNA198412
Sen2Col2	*Bacillus anthracis*	-	5.17411	-	PRJNA198413
Sen3	*Bacillus anthracis*	-	5.17379	-	PRJNA198414
Gmb1	*Bacillus anthracis*	-	5.17508	-	PRJNA198415
V770-NP1-R	*Bacillus anthracis*	-	5.16452	-	PRJNA224116
CZC5	*Bacillus anthracis*	-	5.45434	-	PRJNA224116
95014	*Bacillus anthracis*	-	5.33680	1	PRJNA224116
ANSES_08-8_2(	*Bacillus anthracis*	-	5.44071	-	PRJNA224116
ANSES_99-100	*Bacillus anthracis*	-	5.44647	-	PRJNA224116
ANSES_00-82	*Bacillus anthracis*	-	5.43601	-	PRJNA224116
2000031006	*Bacillus anthracis*	-	5.44337	-	PRJNA264742
2000031023	*Bacillus anthracis*	-	5.43554	-	PRJNA224116
2000031027	*Bacillus anthracis*	-	5.44555	-	PRJNA264742
2000031038	*Bacillus anthracis*	-	5.43107	-	PRJNA264742
2000031031	*Bacillus anthracis*	-	5.44930	-	PRJNA264742
2000031039	*Bacillus anthracis*	-	5.44962	-	PRJNA264742
2000031052	*Bacillus anthracis*	-	5.27409	-	PRJNA264742
2000031075	*Bacillus anthracis*	-	5.35337	-	PRJNA264742
2000031709	*Bacillus anthracis*	-	5.44960	-	PRJNA264742
2000031765	*Bacillus anthracis*	-	5.44942	-	PRJNA264742
2000032819	*Bacillus anthracis*	-	5.45031	-	PRJNA264742
2000032879	*Bacillus anthracis*	-	5.44748	-	PRJNA264742
2000032951	*Bacillus anthracis*	-	5.43193	-	PRJNA264742
2000032967	*Bacillus anthracis*	-	5.44337	-	PRJNA264742
2000032968	*Bacillus anthracis*	-	5.29679	-	PRJNA264742
2000032975	*Bacillus anthracis*	-	5.43915	-	PRJNA264742
2000032979	*Bacillus anthracis*	-	5.43321	-	PRJNA264742
2000032989	*Bacillus anthracis*	-	5.44396	-	PRJNA264742
2002013094	*Bacillus anthracis*	-	5.49956	-	PRJNA264742
2000031008	*Bacillus anthracis*	-	5.45101	-	PRJNA264742
2000032832	*Bacillus anthracis*	-	5.44926	-	PRJNA224116

2000032892	*Bacillus anthracis*	-	5.45053	-	PRJNA264742

a: the different insecticidal toxicity of *B. thuringiensis* was divided based on the reported works and serotypes. And *B. cereus* /*B. anthracis* were marked by "-" for no insecticidal toxicity.

b: the draft genome with unkonwn plasmid content were marked by "-".

Table S3 Insecticidal toxicity related genes (ITRGs) in *B. thuringiensis* and some typical *B. cereus* group genomes

ITRGs Groups	ITRGs protein families	ITRGs name	conserved domains (NCBI CDD Accession Number)	*B. thuringiensis*															
				HD-1		YBT-1520		IS5056		MC28		HD-29		YBT-1518		CT-43		HD-789	
				Gene locus tags	Gene number	Gene locus tags	Gene number	Gene locus tags	Gene number	Gene locus tags	Gene number	Gene locus tags	Gene number	Gene locus tags	Gene number	Gene locus tags	Gene number	Gene locus tags	Gene number
Insecticidal and other virulence synergic genes	parasporal crystal protein gene	*cry*	Endotoxin_N (pfam03945), Endotoxin_M (pfam00555), Endotoxin_mid (pfam09131), Endotoxin_C (pfam03944), delta_endotoxin_C (cd04085)	BTK_32551,cry1Aa; BTK_34176,cry1Ab; BTK_33446,cry1Ac; BTK_32556,cry1Ia; BTK_32596,cry2Aa; BTK_32526,cry2Ab	6	YBT1520_32486,cry1Aa; YBT1520_33311,cry1Ac; YBT1520_32491,cry1Ia; YBT1520_32531,cry2Aa; YBT1520_32461,cry2Ab	5	H175_285p288, H175_285p289, H175_285p297, H175_285p281, H175_107p005, H175_63p05	6	MC28_C001, MC28_C067, MC28_C076, MC28_D165, MC28_E074, MC28_E095, MC28_E038, MC28_E085, MC28_E064	9	BT4G5_30055, BT4G5_30075, BT4G5_32465, BT4G5_32490, BT4G5_32495, BT4G5_32525, BT4G5_33165	7	pBMB0228_00050, pBMB0228_00090, YBT1518_04070	3	CT43_P281270, CT43_P281271, CT43_P281278, CT43_P281265, CT43_P127021	5	BTF1_32366, BTF1_32046, BTF1_32391, BTF1_32386, BTF1_32101, BTF1_31831, BTF1_31826	7
		cyt	Bac_thur_toxin (pfam01338), RICIN (cd00161)	N/A	0	N/A	0	N/A	0	MC28_E051, MC28_E053	2	N/A	0	N/A	0	N/A	0	BTF1_32411, BTF1_32051, BTF1_32111	3
		vip	Binary_toxB (pfam03495), VIP2 (cd00233), Vip3A_N (pfam12495)	BTK_32511,vip3Aa	1	YBT1520_32446,vip3Aa	1	H175_285p276, H175_328p046, H175_328p045	3	N/A	0	BT4G5_32450	1	N/A	0	CT43_P281262	1	N/A	0
		Zwittermycin A gene cluster[a]	N/A[b]	BTK_29464-BTK_29609,zmaA-zmaY	1	YBT1520_29679-YBT1520_29824,zmaA-zmaY	1	H175_233p014-H175_233p050	1	N/A	0	BT4G5_29615-BT4G5_29760	1	N/A	0	N/A	0	N/A	0
		Thuringiensin gene cluster[a]	N/A[b]	N/A	0	N/A	0	H175_107p014-H175_107p028	1	N/A	0	N/A	0	N/A	0	CT43_P127037-CT43_P127041	1	N/A	0
Pathogenic and virulence assistant genes		*parasporin-2* like*	N/A	BTK_29949,pft	1	YBT1520_30154,pft	1	H175_328p105	1	MC28_G067	1	N/A	0	N/A	0	N/A	0	N/A	0
		putative mosquitocidal toxin	RICIN (cd00161), NPP1 (pfam05630), ETX_MTX2 (pfam03318), Toxin_10 (pfam05431)	BTK_31084,nep1	1	YBT1520_31234,nep1	1	N/A	0	MC28_E164, MC28_E086, MC28_F002, MC28_E157	4	N/A	0	N/A	0	N/A	0	N/A	0
	hemolysin	HBL III operon[a]	Bacillus_HBL (pfam05791)	BTK_32381-BTK_32391,hbl III 1; BTK_13600-BTK_13610,hbl III 2; BTK_14140-BTK_14155,hbl III 3	3	YBT1520_32316-YBT1520_32326,hbl III 1; YBT1520_13455-YBT1520_13465,hbl III 2; YBT1520_14010-YBT1520_14025,hbl III 3	3	H175_285p243-H175_285p245, H175_ch2474-H175_ch2477	2	MC28_1608-MC28_1610, MC28_1712-MC28_1714	2	BT4G5_32330-BT4G5_32340, BT4G5_13525-BT4G5_13530, BT4G5_16580-BT4G5_16595	3	YBT1518_13680-YBT1518_13695	1	CT43_CH2433-CT43_CH2436, CT43_P281237-CT43_P281239	2	BTF1_13050-BTF1_13065	1
		Nhe operon	Bacillus_HBL (pfam05791)	BTK_10375-BTK_10385,nhe	1	YBT1520_10215-YBT1520_10225,nhe	1	H175_ch1768-H175_ch1770	1	MC28_1060-MC28_1062	1	BT4G5_10480-BT4G5_10490	1	YBT1518_10495-YBT1518_10505	1	CT43_CH1742-CT43_CH1744	1	BTF1_06780-BTF1_06790	1
		hemolysin III	hlyIII (TIGR01065)	BTK_12310,hly III 1; BTK_28925,hly III 2	2	YBT1520_12135,hly III 1; YBT1520_29050,hly III 2	2	H175_ch2193, H175_ch5585	2	MC28_1417, MC28_4684	2	BT4G5_12200, BT4G5_28935	2	YBT1518_12215, YBT1518_30620	2	CT43_CH2161,	2	BTF1_08490, BTF1_25750	2
		hemolysin A	tly (TIGR00478)	BTK_22060,tlyA	1	YBT1520_22210,tlyA	1	H175_ch4258	1	MC28_3468	1	BT4G5_21740	1	YBT1518_23075	1	CT43_CH4190	1	BTF1_19280	1
		cytolysin K	Leukocidin (pfam07968)	BTK_06380,cytK	1	YBT1520_06330,cytK	1	H175_ch1061, H175_ch3518	2	N/A	0	BT4G5_06955	1	YBT1518_19310	1	CT43_CH1046, CT43_CH2161	2	BTF1_15035, BTF1_15355	2
		xhlA	XhlA (pfam10779)	BTK_30654,xhlA1; BTK_01645,xhlA2; BTK_17365,xhlA3; BTK_18230,xhlA4; BTK_25885,xhlA5	5	YBT1520_30809,xhlA1; YBT1520_01800,xhlA2; YBT1520_17375,xhlA3; YBT1520_18235,xhlA4; YBT1520_25986,xhlA5	5	H175_233p055, H175_ch2639, H175_ch3447, H175_ch5033	4	MC28_2611, MC28_2984, MC28_3155, MC28_5209, MC28_5210	5	BT4G5_01245, BT4G5_13545, BT4G5_17930, BT4G5_30785	4	YBT1518_09185, YBT1518_18795, YBT1518_23620, YBT1518_23625, YBT1518_27220, YBT1518_28665, YBT1518_31675, YBT1518_33227, YBT1518_34746, YBT1518_34751	11	N/A	0	BTF1_14580, BTF1_15100, BTF1_15670, BTF1_19790	4
		alveolysin	Thiol_cytolysin (pfam01289)	BTK_26945,alo	1	YBT1520_27015,alo	1	H175_ch5231	1	MC28_G198, MC28_2437	2	BT4G5_26930	1	YBT1518_28260	1	CT43_CH5149	1	BTF1_23990	1
		phospholipases*	Zn_dep_PLPC (cd11009), sphingomy (TIGR03395), Pat_ExoU_VipD_like (cd07207), PI-PLCc_BcPLC_like (cd08586), Pat_NTE_like_bacteria (cd07228), Hydrolase_4 (pfam12146), Pat_hypo_Ecoli_yjju_like (cd07208)	BTK_03835,plc; BTK_03840,spl; BTK_04380,pla1; BTK_10905,pla2; BTK_16295,pla3; BTK_17305,pla4; BTK_20735,pla5; BTK_18535,plcA; BTK_24985,ytpA1; BTK_10305,ytpA2; BTK_33156,plcD; BTK_07165,lypla1;BTK_10305,lypla2	13	YBT1520_03735,plc; YBT1520_03740,spl; YBT1520_04355,pla1; YBT1520_10765,pla2; YBT1520_16260,pla3; YBT1520_17315,pla4; YBT1520_20865,pla5; YBT1520_18545,plcA; YBT1520_25110,ytpA1; YBT1520_10140,ytpA2; YBT1520_33086,plcD; YBT1520_06950,lypla1; YBT1520_10140,lypla2;	13	H175_328p198, H175_ch0605, H175_ch0606, H175_ch0729, H175_ch1971, H175_ch2490, H175_ch2732, H175_ch3502, H175_ch3764, H175_ch3990, H175_ch4858	11	MC28_C068, MC28_E138, MC28_E156, MC28_E158, MC28_F101, MC28_5401, MC28_5402, MC28_0024, MC28_1042, MC28_1195, MC28_1897, MC28_3212, MC28_1721, MC28_4045, MC28_C065, MC28_E138, MC28_E158, MC28_F101	18	BT4G5_03220, BT4G5_03225, BT4G5_11010, BT4G5_24735, BT4G5_05045, BT4G5_13600, BT4G5_14550, BT4G5_18240	8	YBT1518_04135,YBT1518_04140, YBT1518_04740, YBT1518_10335, YBT1518_11075, YBT1518_14905, YBT1518_19170, YBT1518_20640, YBT1518_26330	9	CT43_CH0600, CT43_CH1106, CT43_CH1722, CT43_CH3446, CT43_CH3928, CT43_CH4780	6	BTF1_01090, BTF1_01095, BTF1_07335, BTF1_01435, BTF1_14915, BTF1_17935, BTF1_22325	7
		immune inhibitor A	Peptidase_M6 (pfam05547)	BTK_03795,inhA1; BTK_07715,inhA2; BTK_14800,inhA3	3	YBT1520_03695,inhA1; YBT1520_07515,inhA2 YBT1520_14685,inhA3	3	H175_ch0600, H175_ch1229, H175_ch3037, H175_ch3131	4	MC28_5394, MC28_G179, MC28_0228, MC28_0512, MC28_2289	5	BT4G5_03190, BT4G5_04255, BT4G5_07865, BT4G5_15980,	4	YBT1518_04100, YBT1518_07435, YBT1518_16485, YBT1518_16965	4	CT43_CH0595, CT43_CH1214, CT43_CH2986,	3	BTF1_01060, BTF1_04125, BTF1_12450	3
Saprophytic and colonization	bacteriocins	Thurin gene cluster[a]*	N/A[b]	BTK_29584-BTK_29594,thrA-thrT	1	YBT1520_29799-YBT1520_29809,thrA-thrT	1	H175_233p016-H175_233p018	1	N/A	0	BT4G5_29735-BT4G5_29745	1	N/A	0	N/A	0	N/A	0
		Thuricin gene cluster[a]	N/A[b]	BTK_29689-BTK_29739,thnP-thnI	1	YBT1520_29904-YBT1520_29949,thnP-thnI	1	N/A	0	N/A	0	N/A	0	N/A	0	N/A	0	N/A	0
		enhancins	Enhancin (pfam03272)	BTK_17920,bel1; BTK_18635,bel2	2	YBT1520_17930,bel1; YBT1520_18685,bel2	2	H175_ch3365, H175_ch3511, H175_ch3512	3	MC28_F084, MC28_F124, MC28_F125, MC28_G152, MC28_2523	5	BT4G5_17640	1	YBT1518_18345	1	CT43_CH3312, CT43_CH3454	2	N/A	0
		chitin binding protein genes*	Chitin_bind_3 (pfam03067)	BTK_15640,chb1; BTK_15815,chb2; BTK_18540,chb3; BTK_27045,chb4	4	YBT1520_15540,chb1; YBT1520_15745,chb2; YBT1520_18550,chb3; YBT1520_27075,chb4	4	H175_ch2836, H175_ch2869, H175_ch3503	3	MC28_1720, MC28_1985, MC28_2013	3	BT4G5_15015, BT4G5_15175, BT4G5_18245	3	YBT1518_15415, YBT1518_15585, YBT1518_19175	3	CT43_CH2786, CT43_CH2820, CT43_CH3447	3	BTF1_11665, BTF1_14920	2
		chitinases	GH18_chitinase (cd06548),GH18_chitinase_D-like	BTK_02625,chi1; BTK_19725,chi2	2	YBT1520_02530,chi1; YBT1520_19825,chi2	2	H175_ch0372, H175_ch3723	2	MC28_F106, MC28_2888, MC28_5109	3	BT4G5_01990, BT4G5_19415	2	YBT1518_02395, YBT1518_20435,	2	CT43_CH0372, CT43_CH3361, CT43_CH3664	3	BTF1_11505, BTF1_16665, BTF1_28050	3
		camelysin	Peptidase_M73 (pfam12389)	BTK_07690,calY1; BTK_07700,calY2; BTK_30449,calY3	3	YBT1520_07490,calY1; YBT1520_07500,calY2	2	H175_ch1224, H175_ch1226, H175_ch4978, H175_85p034	4	MC28_G023, MC28_0507, MC28_0509, MC28_4158, MC28_5364	5	BT4G5_07840, BT4G5_07850	2	YBT1518_07410, YBT1518_07420, YBT1518_32782, YBT1518_26955	4	CT43_CH1209, CT43_CH1211, CT43_CH4894, CT43_P83055	4	BTF1_04100, BTF1_04110	2
		bacillolysins (neutral protease)*	M4_neutral_protease (cd09597),M36 (cd09596)	BTK_03405,npr1; BTK_12150,npr2; BTK_16140,npr3; BTK_17015,npr4; BTK_17915,npr5; BTK_26710,npr6; BTK_28405,npr7	7	YBT1520_03330,npr1; YBT1520_11955,npr2; YBT1520_16080,npr3; YBT1520_17015,npr4; YBT1520_17925,npr5; YBT1520_26780,npr6; YBT1520_28550,npr7;	7	H175_328p006, H175_328p121, H175_233p081, H175_ch0525, H175_ch1771, H175_ch2145, H175_ch2530, H175_ch2770, H175_ch3364, H175_ch5182, H175_ch5482	11	MC28_1391, MC28_1715, MC28_1759, MC28_1930, MC28_2522, MC28_4305, MC28_4587, MC28_5326	8	BT4G5_02870, BT4G5_03925, BT4G5_09185, BT4G5_13890, BT4G5_14695, BT4G5_17635, BT4G5_26715, BT4G5_28425, BT4G5_29800	9	YBT1518_15085, YBT1518_18340, YBT1518_30095, YBT1518_33971, YBT1518_03175, YBT1518_14120	6	CT43_CH0522, CT43_CH2114, CT43_CH2490, CT43_CH2720, CT43_CH3311, CT43_CH5102, CT43_CH5391	7	BTF1_00720, BTF1_10040, BTF1_11185, BTF1_23740, BTF1_25250	5
		subtilisins*	Peptidases_S8_11 (cd04843),Peptidases_S8_Subtilisin_subset (cd07477),Peptidases_S8_Thermitase_like (cd07484)	BTK_30454,sub1; BTK_30864,sub2; BTK_10945,sub3; BTK_12915,sub4; BTK_18005,sub5; BTK_18625,sub6; BTK_19905,sub7	7	YBT1520_31009,sub2; YBT1520_10805,sub3; YBT1520_12745,sub4; YBT1520_18015,sub5; YBT1520_18675,sub6; YBT1520_20000,sub7	6	H175_328p124, H175_328p171, H175_328p174, H175_328p188, H175_233p100, H175_ch1980, H175_ch2313, H175_ch3398, H175_ch3765, H175_ch4979	10	MC28_F086, MC28_G297, MC28_G310, MC28_1224, C28_1586, MC28_2558, MC28_2931, MC28_4159, MC28_5363	9	BT4G5_30735, BT4G5_31005	2	YBT1518_02065, YBT1518_12830, YBT1518_20655, YBT1518_26960, YBT1518_34191,	5	CT43_CH1954, CT43_CH3345, CT43_CH3703, CT43_CH4372, CT43_CH4895,	5	BTF1_20385, BTF1_07385, BTF1_09085, BTF1_14350, BTF1_16865, BTF1_25175	6
		collagenases*	Peptidase_M9_N (pfam08453),Peptidase_M9 (pfam01752),COG0826 (COG0826)	BTK_03160,colA1; BTK_14070,colA2; BTK_14655,colA3; BTK_18690,colA4; BTK_23100,colA5; BTK_23105,colA6	6	YBT1520_03075,colA1; YBT1520_13940,colA2; YBT1520_14525,colA3; YBT1520_18750,colA4; YBT1520_23260,colA5; YBT1520_23265,colA6	6	H175_328p153, H175_ch0479, H175_ch3057, H175_ch3177, H175_ch3526, H175_ch4467, H175_ch4468	7	MC28_2207, MC28_2678, MC28_3672, MC28_3673, MC28_5277	5	BT4G5_02625, BT4G5_03690, BT4G5_16125, BT4G5_16665, BT4G5_17720, BT4G5_18405, BT4G5_19595	7	YBT1518_02940, YBT1518_13825, YBT1518_16590, YBT1518_18525, YBT1518_24360, YBT1518_24365, YBT1518_32215	7	CT43_CH0476, CT43_CH3007, CT43_CH3123, CT43_CH3469, CT43_CH4398, CT43_CH4399	6	BTF1_00485, BTF1_15395, BTF1_20515, BTF1_20520	4
		pectate lyase*	PL-6 (cd14251)	BTK_30289,pel1; BTK_13595,pel2	2	YBT1520_30479,pel1; YBT1520_13450,pel2	2	N/A	0	N/A	0	N/A	0	N/A	0	N/A	0	N/A	0
		cell wall hydrolases*	Lysozyme_like (pfam13702),Peptidase_M23 (pfam01551),Peptidase_S9 (pfam00326)	BTK_29989,cwl1; BTK_30669,cwl2; BTK_30674,cwl3; BTK_34126,cwl4; BTK_05470,cwl5	5	YBT1520_30194,cwl1; YBT1520_30819,cwl2; YBT1520_30824,cwl3; YBT1520_33946,cwl4; YBT1520_05435,cwl5	5	H175_285p104, H175_233p057, H175_233p058, H175_85p062, H175_68p67, H175_ch0908, H175_63p15	6	MC28_C041, MC28_G171, MC28_1080	3	BT4G5_14045, BT4G5_28630	2	YBT1518_20670, YBT1518_04635, YBT1518_08420, YBT1518_10455, YBT1518_27650	5	CT43_CH0702, CT43_CH1407, CT43_CH1734, CT43_CH5033	4	BTF1_16515, BTF1_04860, BTF1_01335, BTF1_05100, BTF1_06745, BTF1_23420, BTF1_01335	7
		Extracellular metalloproteases*	COG3591 (COG3591),SprT (COG3091),M32_Taq (cd06460),Zn_peptidase_2 (pfam04298)	BTK_04505,mpr1; BTK_01280,mpr2; BTK_08940,mpr3; BTK_09125,mpr4	4	YBT1520_04470,mpr1; YBT1520_01210,mpr2; YBT1520_08755,mpr3; YBT1520_08945,mpr4	4	H175_ch0232, H175_ch1473, H175_ch1508	3	MC28_0815, MC28_4945	2	BT4G5_00870, BT4G5_05155, BT4G5_09100, BT4G5_09275	4	YBT1518_01210, YBT1518_08670, YBT1518_08850	3	CT43_CH0231, CT43_CH1455, CT43_CH1490	3	BTF1_01535, BTF1_27225, BTF1_05345, BTF1_05520	4
		aiiA	Lactamase_B (smart00849)	BTK_18210,aiiA	1	YBT1520_18215,aiiA	1	H175_ch3442	1	MC28_2602	1	BT4G5_17910	1	YBT1518_18760	1	CT43_CH3389	1	BTF1_14555	1
	S-layer/cell wall anchor functional protein genes*	internalin	SLH (pfam00395)/LPXTG_anchor (TIGR01167)/choice_anch_A (TIGR04215)/TQXA_dom+NEAT (cd06920)	BTK_03150,ilsA1; BTK_07945,ilsA2; BTK_23860,ilsA3	3	YBT1520_03065,ilsA1; YBT1520_07750,ilsA2; YBT1520_24020,ilsA3	3	H175_ch0476, H175_ch1275, H175_ch4640	3	MC28_2291, MC28_5275, MC28_3827	3	BT4G5_02615, BT4G5_03680, BT4G5_08095	3	YBT1518_02925, YBT1518_07665, YBT1518_08990, YBT1518_09260, YBT1518_19455	5	CT43_CH0473, CT43_CH1261, CT43_CH4567	3	BTF1_00475, BTF1_04355	2
		collagen adhesion protein	SLH (pfam00395)/LPXTG_anchor (TIGR01167)/choice_anch_A (TIGR04215)/TQXA_dom+Cna_B (pfam05738)/Collagen_bind (pfam05737)/RrgB_K2N_iso_D2 (TIGR04226)	BTK_04990,scolB1; BTK_06115,scolB2; BTK_16995,scolB3; BTK_17005,scolB4; BTK_18670,scolB5;	5	YBT1520_04965,scolB1; YBT1520_06060,scolB2; YBT1520_16985,scolB3; YBT1520_16995,scolB4; YBT1520_18720,scolB5	5	H175_ch0851, H175_ch1026, H175_ch2532, H175_ch2534, H175_ch3522	5	MC28_C025, MC28_E018, MC28_0144, MC28_0293, MC28_1763, MC28_1761, MC28_2671	7	BT4G5_05640, BT4G5_13910, BT4G5_18385, BT4G5_06685, BT4G5_13900, BT4G5_24290, BT4G5_26825,	7	YBT1518_05410, YBT1518_03720, YBT1518_14135, YBT1518_14145, YBT1518_19335, YBT1518_30125, YBT1518_32982, YBT1518_31925	8	CT43_CH0841, CT43_CH1013, CT43_CH2492, CT43_CH2494, CT43_CH3465, CT43_CH5397	6	BTF1_01990, BTF1_03060, BTF1_10050, BTF1_10060, BTF1_15375, BTF1_23850, BTF1_25280	7
		peptidoglycan recognition and hydrolyzation	SLH (pfam00395)/LPXTG_anchor (TIGR01167)/choice_anch_A (TIGR04215)/TQXA_dom+PGRP (cd06583)/MurNAc-LAA (cd02696)/Spr (COG0791)/Glucosaminidase (pfam01832)/Transglut_core (pfam01841)	BTK_29644,scwl1; BTK_30509,scwl2; BTK_04995,scwl3; BTK_05035,scwl4; BTK_05065,scwl5; BTK_05725,scwl6; BTK_13645,scwl7; BTK_17350,scwl8	8	YBT1520_29859,scwl1; YBT1520_30669,scwl2; YBT1520_04970,scwl3; YBT1520_05010,scwl4; YBT1520_05040,scwl5; YBT1520_05665,scwl6; YBT1520_13500,scwl7; YBT1520_17360,scwl8	8	H175_ch3519, H175_ch0852,H175_ch0934, H175_ch0860, H175_ch0867, H175_ch3259, H175_ch5353	7	N/A	0	BT4G5_29805, BT4G5_05645, BT4G5_17090	3	YBT1518_17465, YBT1518_05415, YBT1518_17795, YBT1518_05455	4	CT43_CH0842, CT43_CH3204, CT43_CH0851, CT43_CH0923	4	BTF1_01995, BTF1_02065, BTF1_13705, BTF1_15360, BTF1_16425, BTF1_24350,	6
		other function	SLH (pfam00395)/LPXTG_anchor (TIGR01167)/choice_anch_A (TIGR04215)/TQXA_dom+Fib_alpha (pfam08702)/ISOPREN_C2_like (cd00688)/MPP_CpdB_N (cd07410),5_nucleotid_C (pfam02872)/B_ant_repeat (TIGR01451)	BTK_27710,scwlJ; BTK_13745,sfga; BTK_17585,siso; BTK_21775,scpd	4	YBT1520_27900,scwlJ; YBT1520_13610,sfga; YBT1520_17600,siso; YBT1520_21935,scpd	4	H175_ch3234, H175_ch3287, H175_ch4197	3	MC28_4469, MC28_2377, MC28_2450, MC28_3415	4	BT4G5_27830, BT4G5_16995, BT4G5_17305, BT4G5_21450	4	YBT1518_29465, YBT1518_17680, YBT1518_17935, YBT1518_22805	4	CT43_CH5264, CT43_CH3179, CT43_CH4130	3	BTF1_24545, BTF1_13570, BTF1_13960, BTF1_18935	4
Total					105		102		109		110		85		92		79		85

Table S3 Continued.

ITRGs Groups	ITRGs protein families	ITRGs name	conserved domains (NCBI CDD Accession Number)	*Bacillus thuringiensis*													
				HD1002		HD-571		HD-682		HD-771		HD1011		HD-73		407 cry-	
				Gene locus tags	Gene number	Gene locus tags	Gene number	Gene locus tags	Gene number	Gene locus tags	Gene number	Gene locus tags	Gene number	Gene locus tags	Gene number	Gene locus tags	Gene number
Insecticidal and other virulence synergic genes	parasporal crystal protein gene	*cry*	Endotoxin_N (pfam03945), Endotoxin_M (pfam00555), Endotoxin_mid (pfam09131), Endotoxin_C (pfam03944), delta_endotoxin_C (cd04085)	AS86_6831, AS86_6832	2	N/A	0	N/A	0	BTG_32568	1	N/A	0	HD73_6004	1	N/A	0
		cyt	Bac_thur_toxin (pfam01338), RICIN (cd00161)	N/A	0	N/A	0	N/A	0	N/A	0	N/A	0	N/A	0	N/A	0
		vip	Binary_toxB (pfam03495), VIP2 (cd00233), Vip3A_N (pfam12495)	N/A	0	N/A	0	N/A	0	N/A	0	N/A	0	N/A	0	N/A	0
		Zwittermycin A gene cluster[a]	N/A[b]	N/A	0	N/A	0	N/A	0	N/A	0	N/A	0	N/A	0	N/A	0
		Thuringiensin gene cluster[a]	N/A[b]	N/A	0	N/A	0	N/A	0	N/A	0	N/A	0	N/A	0	N/A	0
		parasporin-2 like*	N/A	N/A	0	N/A	0	N/A	0	N/A	0	N/A	0	N/A	0	N/A	0
		putative mosquitocidal toxin	RICIN (cd00161), NPP1 (pfam05630), ETX_MTX2 (pfam03318), Toxin_10 (pfam05431)	AS86_5750	1	N/A	0	N/A	0	N/A	0	N/A	0	N/A	0	N/A	0
		HBL III operon[a]	Bacillus_HBL (pfam05791)	AS86_891-AS86_894, AS86_2126-AS86_2128	2	BF32_746-BF32_749, BF32_4961-BF32_4963	2	BF36_1937-BF36_1940, BF36_3121-BF36_3123	2	BTG_03700-BTG_03715, BTG_11195-BTG_11205	2	BF38_4299-BF38_4302, BF38_3108-BF38_3110	2	HD73_2742-HD73_2744, HD73_2852-HD73_2856	2	BTB_c18590-BTB_c18610, BTB_c25510-BTB_c25540	2
		Nhe operon	Bacillus_HBL (pfam05791)	N/A	0	N/A	0	N/A	0	BTG_11385-BTG_11395	1	N/A	0	HD73_2051-HD73_2053	1	N/A	0
		hemolysinIII	hlyIII (TIGR01065)	AS86_1794, AS86_4055	2	BF32_3048, BF32_5318	2	BF36_2795, BF36_4992	2	BTG_08905, BTG_21120	2	BF38_1172, BF38_3445	2	HD73_2482, HD73_5864	2	BTB_c22730, BTB_c56500	2
		hemolysinA	tly (TIGR00478)	AS86_5356	1	BF32_1773	1	BF36_849	1	BTG_28515	1	BF38_5344	1	HD73_4478	1	BTB_c43170	1
		cytolysinK	Leukocidin (pfam07968)	AS86_436, AS86_502	2	BF32_181	1	BF36_2512, BF36_3833	2	BTG_01835	1	BF38_2340, BF38_3715	2	HD73_1267	1	BTB_c11620, BTB_c35930	2
Pathogenic and virulence assistant genes	hemolysin	*xhlA*	XhlA (pfam10779)	AS86_373, AS86_489, AS86_589, AS86_5255	4	BF32_1076	1	BF36_1375, BF36_1607, BF36_5453	3	BTG_02290, BTG_10425, BTG_28185	3	BF38_5554, BF38_4624, BF38_4704	3	HD73_0298, HD73_3529, HD73_3701, HD73_5245	4	BTB_c27220, BTB_c35270, BTB_c51140	3
		alveolysin	Thiol_cytolysin (pfam01289)	AS86_4399	1	BF32_925	1	BF36_1760	1	BTG_23145	1	BF38_4470	1	HD73_5449	1	BTB_c53120	1
		phospholipases*	Zn_dep_PLPC (cd11009), sphingomy (TIGR03395), Pat_ExoU_VipD_like (cd07207), PI-PLCc_BcPLC_like (cd08586), Pat_NTE_like_bacteria (cd07228), Hydrolase_4 (pfam12146), Pat_hypo_Ecoli_yjju_like (cd07208)	AS86_525, AS86_1301, AS86_3266, AS86_3265, AS86_1882, AS86_2018, AS86_2197, AS86_3172, AS86_4753, AS86_5624,AS86_6837, AS86_5847	12	BF32_342, BF32_1529, BF32_2367, BF32_3814, BF32_3815, BF32_3904, BF32_4879, BF32_5064	8	BF36_259, BF36_1098, BF36_2353, BF36_2913, BF36_3026, BF36_3202, BF36_4155, BF36_4242, BF36_4243	9	BTG_06205, BTG_10000, BTG_17770, BTG_17775, BTG_25085, BTG_29795	6	BF38_518, BF38_1922, BF38_1923, BF38_2011, BF38_2964, BF38_3205, BF38_3326, BF38_3874, BF38_5097	9	HD73_0751, HD73_0752, HD73_1401, HD73_2037, HD73_2158, HD73_3516, HD73_3759, HD73_4047, HD73_4211, HD73_5064	10	BTB_c06910, BTB_c06920, BTB_c20590, BTB_c49120	4
		immune inhibitor A	Peptidase_M6 (pfam05547)	AS86_1010, AS86_2653, AS86_3272	3	BF32_4427, BF32_3810	2	BF36_3668, BF36_4247	2	BTG_04410, BTG_14320, BTG_17805	3	BF38_1918, BF38_2504	2	HD73_0744, HD73_1513, HD73_2986	3	BTB_c06870, BTB_c13280, BTB_c31140	3
	bacteriocins	Thurin gene cluster[a]*	N/A[b]	N/A	0	N/A	0	N/A	0	N/A	0	N/A	0	N/A	0	N/A	0
		Thuricin gene cluster[a]	N/A[b]	N/A	0	N/A	0	N/A	0	N/A	0	N/A	0	N/A	0	N/A	0
		enhancins	Enhancin (pfam03272)	N/A	0	N/A	0	BF36_1676	1	N/A	0	BF38_4553	1	HD73_3642, HD73_3788	2	BTB_c34440	1
		chitin binding protein genes*	Chitin_bind_3 (pfam03067)	AS86_524, AS86_1165, AS86_1197	3	BF32_431, BF32_460	2	BF36_2236, BF36_2268	2	BTG_05205	1	BF38_3995, BF38_3964	2	HD73_3152, HD73_3189, HD73_3760	3	BTB_c29110, BTB_c29450, BTB_c35800	3
		chitinases	GH18_chitinase (cd06548),GH18_chitinase_D-like (cd02871),GH18 PF-ChiA-like (cd06543)	AS86_173, AS86_3514,	2	BF32_1322, BF32_3558	2	BF36_1297, BF36_4488	2	BTG_00780, BTG_05395, BTG_18940	3	BF38_1678, BF38_4896	2	HD73_0514, HD73_1112, HD73_3674, HD73_4009	4	BTB_c04440, BTB_c37960	2
		camelysin	Peptidase_M73 (pfam12389)	AS86_2658, AS86_2656, AS86_5876	3	BF32_2485, BF32_3774, BF32_4423, BF32_4425	4	BF36_3670, BF36_3672	2	BTG_14350, BTG_14340	2	BF38_2500, BF38_2502, BF38_3244	3	HD73_1508, HD73_1510	2	BTB_c13230, BTB_c13250, BTB_c50310	3
		bacillolysins (neutral protease)*	M4_neutral_protease (cd09597),M36 (cd09596)	AS86_1491, AS86_3338, AS86_4448, AS86_1262, AS86_4155	5	BF32_214, BF32_857, BF32_2665, BF32_3737, BF32_5256	5	BF36_2475, BF36_4319, BF36_5357, BF36_2856	4	BTG_06020, BTG_06920, BTG_18155, BTG_21620, BTG_23400	5	BF38_1848, BF38_3750, BF38_801, BF38_3390	4	HD73_0673, HD73_2450, HD73_3261, HD73_3451, HD73_3641, HD73_5406, HD73_5761	7	BTB_c06140, BTB_c22270, BTB_c28480, BTB_c34430, BTB_c52660	5
		subtilisins*	Peptidases_S8_11 (cd04843),Peptidases_S8_Subtilisin_subset (cd07477),Peptidases_S8_Thermitase_like (cd07484)	AS86_134, AS86_634, AS86_4170, AS86_5136, AS86_1677	5	BF32_3773, BF32_2486, BF32_1360, BF32_1950, BF32_2424	5	BF36_672, BF36_1263	2	BTG_27305, BTG_00575, BTG_02525, BTG_08260, BTG_09955	5	BF38_111, BF38_4931, BF38_3565	3	HD73_2167, HD73_2601, HD73_3659, HD73_3786, HD73_4048	5	BTB_c44970, BTB_c50320, BTB_c38350	3
		collagenases*	Peptidase_M9_N (pfam08453),Peptidase_M9 (pfam01752),COG0826 (COG0826)	AS86_428, AS86_3386	2	BF32_1133, BF32_3693	2	BF36_1551, BF36_4363	2	BTG_01795, BTG_18395, BTG_27160, BTG_27165	4	BF38_1804, BF38_4683	2	HD73_0625, HD73_2839, HD73_2953, HD73_3800, HD73_4691, HD73_4692	6	BTB_c05690, BTB_c31330, BTB_c34780, BTB_c36010, BTB_c45220	5
		pectate lyase*	PL-6 (cd14251)	N/A	0	N/A	0	N/A	0	N/A	0	N/A	0	HD73_2741	1	N/A	0
Saprophytic and colonization		cell wall hydrolases*	Lysozyme_like (pfam13702),Peptidase_M23 (pfam01551),Peptidase_S9 (pfam00326)	AS86_202, AS86_203, AS86_1560, AS86_2136, AS86_2508, AS86_3192, AS86_4511, AS86_5706, AS86_5772, AS86_6858, AS86_6748	11	BF32_242, BF32_1361, BF32_2990	3	BF36_50, BF36_3108, BF36_3130, BF36_3520, BF36_4175, BF36_3465	6	BTG_13585, BTG_17515, BTG_23975, BTG_11430, BTG_13345	5	BF38_728, BF38_1991, BF38_2650, BF38_3101, BF38_3123, BF38_4706, BF38_4709, BF38_2698, BF38_4784	7	HD73_1099, HD73_6072	2	BTB_c14730, BTB_c15200, BTB_c08170	3
		Extracellular metalloproteases*	COG3591 (COG3591),SprT (COG3091),M32_Taq (cd06460),Zn_peptidase_2 (pfam04298)	AS86_3713, AS86_6223, AS86_2412, AS86_2377	4	BF32_3378, BF32_4670, BF32_4705	3	BF36_4132, BF36_4661, BF36_3423, BF36_3388	4	BTG_17320, BTG_19705, BTG_13100, BTG_12925	4	BF38_2029, BF38_1504, BF38_5677, BF38_2747, BF38_2782	5	HD73_0235, HD73_0905, HD73_1758, HD73_1796	4	BTB_c02710, BTB_c15690, BTB_c16030	3
		aiiA	Lactamase_B (smart00849)	AS86_594	1	BF32_1068	1	BF36_1612	1	BTG_02315	1	BF38_4619	1	HD73_3697	1	BTB_c35230	1
		internalin	SLH (pfam00395)/LPXTG_anchor (TIGR01167)/choice_anch_A (TIGR04215)/TQXA_dom+NEAT (cd06920)	AS86_3388, AS86_2607	2	BF32_3690, BF32_4473	2	BF36_4367, BF36_3621	2	BTG_14090, BTG_18405, BTG_24125	3	BF38_1800, BF38_2550, BF38_2333	3	HD73_0623, HD73_1560, HD73_4841	3	BTB_c05660, BTB_c13740	2
		collagen adhesion protein	SLH (pfam00395)/LPXTG_anchor (TIGR01167)/choice_anch_A (TIGR04215)/TQXA_dom+Cna_B (pfam05738)/Collagen_bind (pfam05737)/RrgB_K2N_iso_D2 (TIGR04226)	S86_3062, AS86_1487	2	BF32_4194, BF32_2958	2	BF36_4038, BF36_2471, BF36_5084	3	BTG_01815, BTG_06900, BTG_06910, BTG_16870, BTG_23285	5	BF38_2122	1	HD73_1005, HD73_1214， HD73_3446, HD73_3449, HD73_3796	5	BTB_c09570, BTB_c11270, BTB_c11280, BTB_c26110, BTB_c26130, BTB_c35970	6
	S-layer/cell wall anchor functional protein genes*	peptidoglycan recognition and hydrolyzation	SLH (pfam00395)/LPXTG_anchor (TIGR01167)/choice_anch_A (TIGR04215)/TQXA_dom+PGRP (cd06583)/MurNAc-LAA (cd02696)/Spr (COG0791)/Glucosaminidase (pfam01832)/Transglut_core (pfam01841)	AS86_5863, AS86_6170, AS86_6192, AS86_6193, AS86_6749	5	BF32_1282, BF32_180, BF32_903, BF32_2829, BF32_4018, BF32_4040, BF32_4895	7	BF36_1377, BF36_1404, BF36_1781, BF36_2513, BF36_3184, BF36_4013, BF36_4037	7	BTG_00970, BTG_01830, BTG_03140, BTG_08490, BTG_08845, BTG_10410, BTG_11945, BTG_15020, BTG_16790, BTG_16865, BTG_22425, BTG_22975, BTG_23935, BTG_26770	14	BF38_2123, BF38_4454, BF38_4850, BF38_5707, BF38_5708	5	HD73_1006, HD73_1014, HD73_1021, HD73_1135, HD73_2752, HD73_3527	6	BTB_c09580, BTB_c33370, BTB_c35940, BTB_c10380	4
		other function	SLH (pfam00395)/LPXTG_anchor (TIGR01167)/choice_anch_A (TIGR04215)/TQXA_dom+Fib_alpha (pfam08702)/ISOPREN_C2_like (cd00688)/MPP_CpdB_N (cd07410),5_nucleotid_C (pfam02872)/B_ant_repeat (TIGR01451)	AS86_4291, AS86_791, AS86_714, AS86_5424	4	BF32_2829, BF32_839, BF32_937, BF32_1726	4	BF36_5205, BF36_1834, BF36_1748, BF36_897	4	BTG_22225, BTG_03265, BTG_02920, BTG_28790	4	BF38_960, BF38_4401, BF38_4482, BF38_5296	4	HD73_2772, HD73_5638, HD73_3575, HD73_4425	4	BTB_c54270, BTB_c33130, BTB_c33640, BTB_c42590	4
		Total			79		60		64		77		65		81		63

Table S3 Continued.

ITRGs Groups	ITRGs protein families	ITRGs name	conserved domains (NCBI CDD Accession Number)	*Bacillus thuringiensis*								*B. cereus*				*B. anthracis*	
				YBT-020		BMB171		Al Hakam		97-27		ATCC14579		ATCC10987		Ames Ancestor	
				Gene locus tags	Gene number	Gene locus tags	Gene number	Gene locus tags	Gene number	Gene locus tags	Gene number	Gene locus tags	Gene number	Gene locus tags	Gene number	Gene locus tags	Gene number
Insecticidal and other virulence synergic genes	parasporal crystal protein gene	*cry*	Endotoxin_N (pfam03945), Endotoxin_M (pfam00555), Endotoxin_mid (pfam09131), Endotoxin_C (pfam03944), delta_endotoxin_C (cd04085)	YBT020_27999, YBT020_28569, YBT020_29291	3	N/A	0	N/A	0	N/A	0	N/A	0	N/A	0	N/A	0
		cyt	Bac_thur_toxin (pfam01338), RICIN (cd00161)	N/A	0	N/A	0	N/A	0	N/A	0	N/A	0	N/A	0	N/A	0
		vip	Binary_toxB (pfam03495), VIP2 (cd00233), Vip3A_N (pfam12495)	N/A	0	N/A	0	N/A	0	N/A	0	N/A	0	N/A	0	N/A	0
		Zwittermycin A gene cluster[a]	N/A[b]	N/A	0	N/A	0	N/A	0	N/A	0	N/A	0	N/A	0	N/A	0
		Thuringiensin gene cluster[a]	N/A[b]	N/A	0	N/A	0	N/A	0	N/A	0	N/A	0	N/A	0	N/A	0
Pathogenic and virulence assistant genes		*parasporin-2* like*	N/A	N/A	0	N/A	0	N/A	0	N/A	0	N/A	0	N/A	0	N/A	0
		putative mosquitocidal toxin	RICIN (cd00161), NPP1 (pfam05630), ETX_MTX2 (pfam03318), Toxin_10 (pfam05431)	N/A	0	N/A	0	N/A	0	N/A	0	N/A	0	N/A	0	N/A	0
	hemolysin	HBL III operon[a]	Bacillus_HBL (pfam05791)	YBT020_15525-YBT020_15540	1	BMB171_C2812-BMB171_C2815	1	BALH_2803-BALH_2807	1	BT9727_2890-BT9727_2893	1	BC3101-BC3104,	1	N/A	0	N/A	0
		Nhe operon	Bacillus_HBL (pfam05791)	YBT020_09830-YBT020_09840	1	BMB171_C1672-BMB171_C1674	1	BALH_1663-BALH_1665	1	BT9727_1727-BT9727_1729	1	BC1809-BC1811	1	BCE_1968-BCE_1970	1	GBAA_1887-GBAA_1889	1
		hemolysinIII	hlyIII (TIGR01065)	YBT020_11485, YBT020_27210	2	BMB171_C1976, BMB171_C5045	2	BALH_2005, BALH_4959	2	BT9727_2027, BT9727_5132	2	BC2196, BC5449	2	BCE_2271, BCE_5590	2	GBAA_2241, GBAA_5701	2
		hemolysinA	tly (TIGR00478)	YBT020_20610	1	BMB171_C3836	1	BALH_3784	1	BT9727_3918	1	BC4175	1	BCE_4248	1	GBAA_4399	1
		cytolysinK	Leukocidin (pfam07968)	YBT020_17240,	1	BMB171_C0978	1	BALH_2275	1	BT9727_2311	1	BC1110, BC3523	2	BCE_1209	1	N/A	0
		xhlA	XhlA (pfam10779)	YBT020_02040	1	BMB171_C3137, BMB171_C3366	2	N/A	0	N/A	0	BC1909, BC3458, BC3696	3	N/A	0	N/A	0
		alveolysin	Thiol_cytolysin (pfam01289)	YBT020_16250	1	BMB171_C4692	1	BALH_2974	1	BT9727_3096	1	BC5101	1	BCE_3314	1	GBAA_3355	1
		phospholipases*	Zn_dep_PLPC (cd11009), sphingomy (TIGR03395), Pat_ExoU_VipD_like (cd07207), PI-PLCc_BcPLC_like (cd08586), Pat_NTE_like_bacteria (cd07228), Hydrolase_4 (pfam12146), Pat_hypo_Ecoli_yjju_like (cd07208)	YBT020_03650, YBT020_03655, YBT020_04000, YBT020_09715, YBT020_10320, YBT020_19295	6	BMB171_C0589,BMB171_C0590, BMB171_C1652, BMB171_C4395,	4	BALH_0617, BALH_0618, BALH_0679, BALH_1636, BALH_1763, BALH_2428, BALH_3556, BALH_4334	8	BT9727_0587, BT9727_0588, BT9727_0653, BT9727_1821, BT9727_2473, BT9727_3669, BT9727_4492,	7	BC0670, BC0671, BC0759, BC1788, BC1989, BC4753,	6	BCE_0744, BCE_0745, BCE_0815, BCE_2070, BCE_3671, BCE_3973, BCE_4900, BCE_4904	8	GBAA_0677, GBAA_0678, GBAA_0745, GBAA_1992, GBAA_2697, GBAA_3703, GBAA_4137, GBAA_5009	8
		immune inhibitor A	Peptidase_M6 (pfam05547)	YBT020_03625, YBT020_07070, YBT020_14920	3	BMB171_C0585, BMB171_C1128, BMB171_C2685, BMB171_C2796	4	BALH_0614, BALH_1145	2	BT9727_0582, BT9727_1175	2	BC0666, BC1284, BC2984	3	BCE_0740, BCE_1396, BCE_3037	3	GBAA_0672, GBAA_1295	2
Saprophytic and colonization	bacteriocins	Thurin gene cluster[a]*	N/A[b]	N/A	0	N/A	0	N/A	0	N/A	0	N/A	0	N/A	0	N/A	0
		Thuricin gene cluster[a]	N/A[b]	N/A	0	N/A	0	N/A	0	N/A	0	N/A	0	N/A	0	N/A	0
		enhancins	Enhancin (pfam03272)	N/A	0	BMB171_C3071	1	N/A	0	BT9727_3171	1	BC3384	1	N/A	0	GBAA_3443	1
		chitin binding protein genes*	Chitin_bind_3 (pfam03067)	YBT020_14125, YBT020_13950	2	BMB171_C2502, BMB171_C2529	2	BALH_2510, BALH_2536	2	BT9727_2556, BT9727_2586	2	BC2798, BC2827	2	BCE_2824, BCE_2855	2	GBAA_2793, GBAA_2827	2
		chitinases	GH18_chitinase (cd06548),GH18_chitinase_D-like (cd02871),GH18_PF-ChiA-like (cd06543)	YBT020_02460, YBT020_18245	2	BMB171_C0370, BMB171_C3398	2	BALH_0385, BALH_3351	2	BT9727_0362, BT9727_3469	2	BC0429, BC3725	2	BCE_0497, BCE_3753	2	GBAA_0385, GBAA_3854	2
		camelysin	Peptidase_M73 (pfam12389)	YBT020_07045, YBT020_07055, YBT020_28686	3	BMB171_C1123, BMB171_C1125, BMB171_C4507	3	BALH_0576, BALH_1141, BALH_1143, BALH_4433	4	BT9727_1171, BT9727_1173	2	BC1279, BC1281, BC4868	3	BCE_1390, BCE_1392, BCE_5030	3	GBAA_1288, GBAA_1290	2
		bacillolysins (neutral protease)*	M4_neutral_protease (cd09597),M36 (cd09596)	YBT020_03270, YBT020_12800, YBT020_13655, YBT020_26695, YBT020_25080	5	BMB171_C0524, BMB171_C1948, BMB171_C2266, BMB171_C2267, BMB171_C2449, BMB171_C3070, BMB171_C4651, BMB171_C4947	8	BALH_0539, BALH_1941, BALH_2309, BALH_2452, BALH_4578	5	BT9727_0509, BT9727_1996, BT9727_2348, BT9727_2499, BT9727_3170, BT9727_4752	6	BC0602, BC2167, BC2506, BC2735, BC3383, BC5036, BC5351	7	BCE_0667, BCE_2242, BCE_2571, BCE_2762, BCE_5183,	5	GBAA_0599, GBAA_1551, GBAA_2183, GBAA_2568, GBAA_2730, GBAA_3442, GBAA_5282	7
		subtilisins*	Peptidases_S8_11 (cd04843),Peptidases_S8_Subtilisin_subset (cd07477),Peptidases_S8_Thermitase_like (cd07484)	YBT020_10360, YBT020_12085, YBT020_18430, YBT020_24025, YBT020_21455	5	BMB171_C4018, BMB171_C4508	2	BALH_1771, BALH_2119, BALH_3389	3	BT9727_0861, BT9727_1829, BT9727_3502	3	BC3412, BC3762, BC4869, BC4351	4	BCE_4437, BCE_5031	2	GBAA_3660, GBAA_3892, GBAA_5710	3
		collagenases*	Peptidase_M9_N (pfam08453),Peptidase_M9 (pfam01752),COG0826 (COG0826)	YBT020_03025, YBT020_17280, YBT020_21585, YBT020_21590	4	BMB171_C0475, BMB171_C2222, BMB171_C2705, BMB171_C2706, BMB171_C3206, BMB171_C3432, BMB171_C4044	7	BALH_0495, BALH_3172, BALH_3966, BALH_3967	4	BT9727_0466, BT9727_3286	2	BC0556, BC2466, BC3009, BC3010, BC3161, BC3529, BC4377	7	BCE_0616, BCE_3539	2	GBAA_0555, GBAA_3299, GBAA_3584	3
		pectate lyase*	PL-6 (cd14251)	N/A	0	N/A	0	N/A	0	N/A	0	N/A	0	N/A	0	N/A	0
		cell wall hydrolases*	Lysozyme_like (pfam13702),Peptidase_M23 (pfam01551),Peptidase_S9 (pfam00326)	YBT020_12945, YBT020_18435, YBT020_26880	3	BMB171_C0634, BMB171_C1267, BMB171_C1313, BMB171_C1665, BMB171_C3368, BMB171_C4584	6	N/A	0	BT9727_2376, BT9727_3503, BT9727_5071	3	BC0740, BC1431, BC1480, BC1802, BC3698, BC4974	6	BCE_3795	1	GBAA_3893	1
		Extracellular metalloproteases*	COG3591 (COG3591),SprT (COG3091),M32_Taq (cd06460),Zn_peptidase_2 (pfam04298)	YBT020_01330, YBT020_08290, YBT020_08470	3	BMB171_C0230, BMB171_C1361, BMB171_C1400	3	BALH_0241, BALH_1382, BALH_1413	3	BT9727_0228, BT9727_1410, BT9727_1443	3	BC0789, BC0268, BC1528, BC1566	4	BCE_0277, BCE_0860, BCE_1693, BCE_1656	4	GBAA_0257, GBAA_1587	2
		aiiA	Lactamase_B (smart00849)	YBT020_16950	1	BMB171_C3134	1	BALH_3106	1	BT9727_3232	1	BC3453	1	BCE_3466	1	GBAA_3514	1
	S-layer/cell wall anchor functional protein genes*	internalin	SLH (pfam00395)/LPXTG_anchor (TIGR01167)/choice_anch_A (TIGR04215)/TQXA_dom+NEAT (cd06920)	YBT020_02995, YBT020_07300, YBT020_22350	3	BMB171_C0472, BMB171_C1174	2	BALH_0491, BALH_1193, BALH_4131	3	BT9727_0463, BT9727_1005, BT9727_1220	3	BC0552, BC1331, BC4547	3	BCE_0607, BCE_1444, BCE_4671	3	GBAA_0552, GBAA_4787	2
		collagen adhesion protein	SLH (pfam00395)/LPXTG_anchor (TIGR01167)/choice_anch_A (TIGR04215)/TQXA_dom+Cna_B (pfam05738)/Collagen_bind (pfam05737)/RrgB_K2N_iso_D2 (TIGR04226)	YBT020_26725, YBT020_24920	2	BMB171_C0765, BMB171_C2268, BMB171_C2270, BMB171_C3203, BMB171_C4812, BMB171_C4953, BMB171_C4954	7	BALH_0943, BALH_4553, BALH_4715, BALH_4855	4	BT9727_5039, BT9727_4726,colB;	2	BC0887, BC1060, BC2508, BC2510, BC3526, BC5358	6	BCE_0961, BCE_5487, BCE_5331, BCE_5153	4	GBAA_0871, GBAA_5258	2
		peptidoglycan recognition and hydrolyzation	SLH (pfam00395)/LPXTG_anchor (TIGR01167)/choice_anch_A (TIGR04215)/TQXA_dom+PGRP (cd06583)/MurNAc-LAA (cd02696)/Spr (COG0791)/Glucosaminidase (pfam01832)/Transglut_core (pfam01841)	YBT020_18030, YBT020_05150, YBT020_04640, YBT020_16165, YBT020_17245, YBT020_04760	6	BMB171_C0773, BMB171_C2217, BMB171_C0766, BMB171_C2929, BMB171_C4829	5	BALH_0788, BALH_0810, BALH_0879, BALH_1594, BALH_1595, BALH_2274, BALH_2955, BALH_3313	8	BT9727_3111, BT9727_3033, BT9727_2310, BT9727_0796, BT9727_0788, BT9727_0900	6	BC0888, BC3257, BC0896, BC2464, BC3524, BC0902, BC0991, BC1953	8	BCE_3710, BCE_0962, BCE_1890, BCE_0984, BCE_1072, BCE_3295,BCE_1890	7	GBAA3737, GBAA1818, GBAA_2528, GBAA_0898, GBAA_pXO2_0045, GBAA_0981	6
		other function	SLH (pfam00395)/LPXTG_anchor (TIGR01167)/choice_anch_A (TIGR04215)/TQXA_dom+Fib_alpha (pfam08702)/ISOPREN_C2_like (cd00688)/MPP_CpdB_N (cd07410),5_nucleotid_C (pfam02872)/B_ant_repeat (TIGR01451)	YBT020_26085, YBT020_16035, YBT020_16310, YBT020_20310	4	BMB171_C4829, BMB171_C2906, BMB171_C2994, BMB171_C3788	4	BALH_4732, BALH_2894, BALH_2985, BALH_3739	4	BT9727_4915,YBT9727_3006, BT9727_3108, BT9727_3864	4	BC5234	1	N/A	0	GBAA_3254, GBAA_3367, GBAA_4346	3
Total					63		70		60		56		75		53		52

a: a gene operon or cluster counted as one gene

b: the gene cluster were predicted according to the published works

*: candidate virulence or pathogenic related genes

Table S4. *B. thuringiensis* YBT-1520 microarray data of ITRGs and other genes mentioned in this study.

The column A-C represent the gene code in YBT-1520, conventional gene name, gene function annotated mainly by NCBI PGAAP, respectively.

The column D-G display the normalized gene expression level values of each gene at 6th, 9th, 18th, and 27th hours, which represent ML, ES, MS, and LS described in Figure 5, respectively.

Gene groups	Gene Code	Gene name	Gene function	6 h-value (ML)	9 h-value (ES)	18 h-value (MS)	27 h-value (LS)
	YBT1520_32486	cry1Aa	Pesticidal crystal protein cry1Aa	969.74	389.87	9810.47	11976.1
	YBT1520_33311	cry1Ac	Pesticidal crystal protein cry1Aa	822.15	252.52	22121.26	0
	YBT1520_32491	cry1Ia	Cry1Ia	1041.28	904.33	531.95	57.66
	YBT1520_32531	cry2Aa	Cry2Aa	754.24	1921.52	20846.7	1243.49
	YBT1520_32461	cry2Ab	Pesticidal crystal protein cry2Ab	379.38	1375.26	2809.93	1001.17
	YBT1520_32446	vip3Aa	vegetative insecticidal protein Vip3V	364.48	651.27	0	0
	YBT1520_29679	zmaA	non-ribosomal peptide synthase/amino acid adenylation enzyme	172.72	8990.89	18.51	0
	YBT1520_29684	zmaB	putative non-ribosomal peptide synthase	298.31	2078.05	0	0
	YBT1520_29689	zmaC	fusaricidin synthetase FusA	58.91	3748.41	0	33.11
	YBT1520_29699	zmaD	hypothetical protein	141.89	0	43.91	6.74
	YBT1520_29699	zmaE	acyl-CoA dehydrogenase, N-terminal domain protein	0	0	0	0
	YBT1520_29704	zmaR	acetyltransferase	734.67	0	1026.33	859.46
	YBT1520_29709	zmaF	malonyl CoA-acyl carrier protein transacylase	538.75	9335.97	219.78	387.7
	YBT1520_29714	zmaG	3-hydroxyacyl-CoA dehydrogenase	378.25	9516.17	84.71	0
Insecticidal and other virulence synergic	YBT1520_29719	zmaH	acyl carrier protein	457.07	8149.4	0	0
	YBT1520_29724	zmaI	acyl-CoA dehydrogenase	0	6225.87	155.78	857.31

genes	YBT1520_29729	zmaJ	amino acid adenylation enzyme/thioester reductase family protein	224.17	6283.27	82.5	0
	YBT1520_29734	zmaK	amino acid adenylation enzyme/thioester reductase family protein	428.92	0	964.78	338.01
	YBT1520_29739	zmaL	alkanesulfonate monooxygenase	311.91	4901.04	989.21	281.56
	YBT1520_29744	zmaM	cyclic peptide transporter	132.53	2676.18	103.02	96.3
	YBT1520_29749	zmaN	methoxymalonyl-ACP biosynthesis protein	189.52	8560.56	282.31	27.59
	YBT1520_29754	zmaO	amino acid adenylation protein	0	1717.86	0	0
	YBT1520_29759	zmaP	lichenysin synthase LchAD	67.92	989.81	24.86	52.13
	YBT1520_29764	zmaQ	tyrocidine synthetase III	597.56	9762.69	44.89	370.82
	YBT1520_29769	zmaS	4'-phosphopantetheinyl transferase	0	1520.28	0	0
	YBT1520_29774	zmaT	ornithine carbamoyltransferase	101.35	7487.21	117.68	0
	YBT1520_29779	zmaU	pyridoxal-phosphate dependent enzyme	0	4480.97	0	0
	YBT1520_29784	zmaV	hypothetical protein	0	0	0	0
	YBT1520_29814	zmaW	ABC transporter	595.39	528.8	827.99	1820.15
	YBT1520_29819	zmaX	hypothetical protein	199.4	270.56	0	0
	YBT1520_29824	zmaY	hypothetical protein	0	9.72	0	0
	YBT1520_29789	thrR	hypothetical protein	171.85	5790.91	58.08	0
	YBT1520_29799	thrA	lantibiotic protein	507.74	5849.1	212.21	93.85
	YBT1520_29804	thrM	Lantibiotic mersacidin modifying enzyme	200.09	0	32.19	47.22
	YBT1520_29809	thrT	transporter	595.91	250.04	50.75	0
	YBT1520_29904	thnP	MutP	415.76	4.65	502.15	444.75
	YBT1520_29909	thnE	ABC transporter permease	321.18	85.84	59.54	30.66
	YBT1520_29914	thnD	ABC transporter ATP-binding protein	310.26	259.39	160.43	0
	YBT1520_29919	thnR	GntR family transcriptional regulator	0	0	0	0

	YBT1520_29924	thnA1	hypothetical protein	0	0	0	0
	YBT1520_29929	thnA2	hypothetical protein	2885.8	8513.42	2117.2	616.52
	YBT1520_29934	thnA3	hypothetical protein	1418.4	6389.05	781.09	1447.77
	YBT1520_29939	thnB	ywiA protein	612.2	682.4	161.65	256.42
	YBT1520_29944	thnT	ABC-type transport system,membrane ATPase component	282.46	110.32	0	0
	YBT1520_29949	thnI	hypothetical protein	0	0	0	0
	YBT1520_30154	pft	Hydralysin-2	0	679.92	8.25	0
	YBT1520_31234	nep1	mosquitocidal toxin protein	0	0	33.65	0
	YBT1520_32316	hblIII1A	hypothetical protein	5796.8	308.79	68.34	0
	YBT1520_32321	hblIII1B	Tripartite hemolysin BL component L1	10688.82	194.78	0	0
	YBT1520_32326	hblIII1C	Hemolysin BL-binding component	0	0	0	0
	YBT1520_13455	hblIII2A	hypothetical protein	6165.17	260.41	19.49	0
	YBT1520_13460	hblIII2B	Tripartite hemolysin BL component L1	1965.79	0	0	118.39
	YBT1520_13465	hblIII2C	Hemolysin BL-binding component	422.34	0	6.05	0
	YBT1520_14010	hblIII3A	hemolysin C	1816.47	248.68	0	0
	YBT1520_14015	hblIII3B	hemolysin BL lytic component L1	8119.86	380.17	0	0
	YBT1520_14020	hblIII3C	Hbl B protein	1295.06	140.54	31.21	0
	YBT1520_14025	hblIII3D	hemolysin BL binding component	0	0	0	0
Pathogenic and virulence assistant genes	YBT1520_10210	nheA	non-hemolytic enterotoxin lytic component L2	0	0	0	0
	YBT1520_10220	nheB	enterotoxin B	7477.53	628.49	462.58	375.13
	YBT1520_10225	nheC	enterotoxin C	3384.53	192.3	2455.02	2223.51
	YBT1520_12135	hlyIII1	hemolysin III	101.18	27.65	2658	250.9
	YBT1520_29050	hlyIII2	hemolysin III	0	0	0	0
	YBT1520_22210	tlyA	hemolysin A	751.73	543.01	150.16	113.78
	YBT1520_06330	cytK	cytotoxin K	628.65	204.03	0	82.81
	YBT1520_30809	xhlA1	XpaF1 protein	179.05	208.76	45.14	27.59
	YBT1520_01800	xhlA2	cof family hydrolase	0	0	0	0
	YBT1520_17375	xhlA3	cof family hydrolase	0	0	0	4214.51

YBT1520_18235	xhlA4	hypothetical protein	663.47	18.4	7.27	19843.22
YBT1520_27015	alo	perfringolysin O precursor	4509.81	2738.21	97.9	0
YBT1520_03735	plc	phospholipase C	42.45	0	0	0
YBT1520_03740	spl	sphingomyelinase C	177.74	57.2	0	0
YBT1520_04355	pla1	phospholipase	181.9	0	2.88	0
YBT1520_10765	pla2	phospholipase, patatin family protein	0	0	296.97	163.17
YBT1520_16260	pla3	serine protease	294.84	70.73	1426.43	38884.04
YBT1520_17315	pla4	patatin phospholipase	0	0	1697.08	105.2
YBT1520_20865	pla5	putative NTE family protein YlbK	676.98	868.92	1029.75	0
YBT1520_18545	plcA	1-phosphatidylinositol phosphodiesterase PlcA	334.17	547.3	0	0
YBT1520_25110	ytpA1	phospholipase YtpA	240.62	600.75	1030.24	733.08
YBT1520_10140	ytpA2	alpha/beta fold family hydrolase	73.63	15.02	245.19	0
YBT1520_33086	plcD	hypothetical protein	552.78	300.56	12.65	0
YBT1520_03695	inhA1	immune inhibitor A metalloprotease	10690.9	2899.47	0	2077.8
YBT1520_07515	inhA2	immune inhibitor A metalloprotease InhA1	348.72	971.66	352.17	0
YBT1520_14685	inhA3	immune inhibitor A	981	1909.79	942.31	1006.69
YBT1520_17930	bel1	metallprotease, enhancin family protein	729.3	0	0	115.93
YBT1520_18685	bel2	hypothetical protein	6.94	281.39	53.19	88.94
YBT1520_15540	chiB1	chitin binding protein	701.24	0	741.03	324.51
YBT1520_15745	chiB2	chitinase	488.69	433.63	0	0
YBT1520_18550	chiB3	chitin-binding protein	361.02	543.24	0	200.59
YBT1520_27075	chiB4	chitin-binding protein	0	0	799.66	14.71
YBT1520_02530	chi1	endochitinase	161.46	2329.98	1276.94	453.34
YBT1520_19825	chi2	exochitinase	0	738.56	38.54	48.45
YBT1520_07490	caly1	hypothetical protein	404.15	0	4085.47	3314.55
YBT1520_07500	caly2	cell envelope-bound metalloprotease (camelysin)	177.4	13941.75	1850.47	853.32
YBT1520_03330	npr1	neutral protease	229.88	8834.37	1895.41	453.95

	YBT1520_11955	npr2	bacillolysin	0	0	0	0
	YBT1520_16080	npr3	metalloendopeptidase	507.05	0	4.34	25.75
	YBT1520_17015	npr4	neutral protease	137.73	0	0	0
	YBT1520_17925	npr5	bacillolysin	756.33	0	55.88	0
	YBT1520_26780	npr6	bacillolysin	1295.58	13802.25	2595.71	842.59
	YBT1520_28550	npr7	bacillolysin	635.06	107.72	0	125.75
	YBT1520_31009	spr1	peptidase S8 and S53 subtilisin kexin sedolisin	842.24	703.82	1459.65	1923.82
	YBT1520_10805	spr2	intracellular serine protease	596.43	10039.43	1477	368.38
	YBT1520_12745	spr3	alkaline serine protease, subtilase	0	0	1320.43	0
	YBT1520_18015	spr4	collagenase	0	81.78	0	1425.69
	YBT1520_18675	spr5	fibronectin type III domain-containing protein	177.75	119	43.91	135.26
	YBT1520_20000	spr6	serine protease, subtilase	1979.3	0	10.21	0
	YBT1520_03075	colA1	microbial collagenase	6059.85	18.97	85.92	0
	YBT1520_13940	colA2	collagenase	123	0	0	0
	YBT1520_14525	colA3	collagenase	42.28	384.91	256.42	0
	YBT1520_18750	colA4	microbial collagenase	230.58	334.16	231.5	15.94
	YBT1520_23260	colA5	protease	3290.46	1179.04	1541.24	100.29
	YBT1520_23265	colA6	peptidase U32	2013.95	548.43	775.72	576.03
	YBT1520_30479	pel1	hypothetical protein	35.44	110.08	638.45	0
	YBT1520_13450	pel2	hypothetical protein	309.4	639.32	4268.17	7.35
Saprophytic and colonization related genes	YBT1520_30194	cwl1	putative hydrolase	817.3	0	108.89	152.74
	YBT1520_30819	cwl2	cell wall endopeptidase, family M23/M37	320.83	100.73	133.8	0
	YBT1520_30824	cwl3	hypothetical protein	0	41.41	64.43	1059.45
	YBT1520_33946	cwl4	TraG	187.96	119.22	40.49	0
	YBT1520_05435	cwl5	hydrolase	124.22	0	0	0
	YBT1520_01210	mpr1	hypothetical protein	135.13	0	0	31.27
	YBT1520_04470	mpr2	extracellular metalloprotease protein	514.15	733.37	1544.17	0

	YBT1520_08755	mpr3	neutral zinc metallopeptidase	1454.95	1000.52	758.13	10.42
	YBT1520_08945	mpr4	thermostable carboxypeptidase 1	4248.24	0	99.6	0
	YBT1520_18215	aiiA	metallo-beta-lactamase family protein	1776.63	6232.07	484.56	411.62
	YBT1520_03065	ilsA1	internalin protein	154.36	0	20.46	25.14
	YBT1520_07750	ilsA2	internalin	0	15.92	828.48	543.52
	YBT1520_24020	ilsA3	NEAT family protein	89.83	165.57	0	0
	YBT1520_04965	scolB1	LPXTG-motif cell wall anchor domain-containing protein	152.63	95.77	0	46.61
	YBT1520_06060	scolB2	collagen adhesion protein	198.88	174.7	350.22	732.47
	YBT1520_16985	scolB3	collagen adhesion protein	363.62	253.42	0	0
	YBT1520_16995	scolB4	collagen adhesion protein	59.78	312.29	0	0
	YBT1520_18720	scolB5	collagen adhesion protein	1140.37	13138.37	262.28	650.88
	YBT1520_29859	scwl1	N-acetylmuramoyl-L-alanine amidase	1503.28	515.16	3769.15	5252.81
	YBT1520_30669	scwl2	S-layer protein / peptidoglycan endo-beta-N-acetylglucosaminidase	220.79	17.73	0	0
	YBT1520_04970	scwl3	N-acetylmuramoyl-L-alanine amidase	2100.39	185.87	87.63	0
	YBT1520_05010	scwl4	S-layer protein/peptidoglycan	3286.13	274.85	127.94	302.43
	YBT1520_05040	scwl5	N-acetylmuramoyl-L-alanine amidase	711.28	207.41	0	0
	YBT1520_05665	scwl6	S-layer-like domain-containing protein	380.16	160.16	315.05	740.14
	YBT1520_13500	scwl7	N-acetylmuramoyl-L-alanine amidase	2120.57	7.13	495.06	578.49
	YBT1520_17360	scwl8	S-layer protein / peptidoglycan endo-beta-N-acetylglucosaminidase	287.05	47.05	0	0
	YBT1520_27900	scwlJ	cell wall hydrolase	4463.47	2664.46	29.01	0
	YBT1520_13610	sfga	surface protein	517.96	457.76	0	0
	YBT1520_17600	siso	hypothetical protein	581.54	694.8	0	0
	YBT1520_21935	scpd	bifunctional 2',3'-cyclic nucleotide 2'-phosphodiesterase/3'-nucleotidase precursor protein	745.58	122.6	78.11	1523.84
	YBT1520_28545	plcr	Transcriptional activator PlcR	354.43	2021.21	55.15	85.87
Global virulence	YBT1520_03320	nprr	transcriptional regulator	3678.84	5371.4	471.37	69.31

regulator genes	YBT1520_20325	codY	transcriptional repressor CodY	5378.54	4088.3	6948.7	338.62
	YBT1520_32436	inpr	hypothetical protein	0	0	0	0
Unique genes in chromosome inversion ends	YBT1520_13365		3-oxoacyl-ACP reductase	0	109.97	0	0
	YBT1520_13370		glycosyl hydrolase	291.21	286.57	49.29	25.14
	YBT1520_13375		hypothetical protein	117.98	511.89	454.27	0
	YBT1520_13380		hypothetical protein	145.18	0	24.86	0
	YBT1520_13385		hypothetical protein	178.09	99.83	49.29	65.63
	YBT1520_13390		beta-lactamase	72.33	111.78	67.6	79.74
	YBT1520_13395		hypothetical protein	0	0	0	0
	YBT1520_13400		lipoprotein	0	0	0	0
	YBT1520_13405		sensor histidine kinase	0	0	0	0
	YBT1520_13410		hypothetical protein	303.51	0	1237.86	0
	YBT1520_13415		Reverse transcriptase / RNA maturase / Endonuclease	2640.34	161.85	7.76	0
	YBT1520_13420		GNAT family acetyltransferase	0	38.59	0	0
	YBT1520_13425		magnesium transporter	0	0	86.9	0
	YBT1520_13430		Mg(2+) transport ATPase, P-type	93.9	88.32	180.21	102.44
	YBT1520_13435		group-specific protein	8200.41	10277.15	1811.39	550.88
	YBT1520_13440		transcriptional repressor PagR	131.84	2248.67	83.97	0
	YBT1520_13445		hypothetical protein	98.92	477.72	392.72	190.16
	YBT1520_13450	pel2	hypothetical protein	309.4	639.32	4268.17	7.35
	YBT1520_13455	hblIII2A	hypothetical protein	6165.17	260.41	19.49	0
	YBT1520_13460	hblIII2B	Tripartite hemolysin BL component L1	1965.79	0	0	118.39
	YBT1520_13465	hblIII2C	Hemolysin BL-binding component	422.34	0	6.05	0
	YBT1520_13470		hypothetical protein	0	0	0	0
	YBT1520_13475		response regulator aspartate phosphatase	134.96	9.83	0	1.21
	YBT1520_13480		DNA replication protein	0	0	0	0
	YBT1520_13485		transposase for insertion sequence element IS232	271.11	200.64	141.62	93.23

	YBT1520_13490		hypothetical protein	126.29	173.35	47.33	150.29
	YBT1520_13495		group-specific protein	149.77	0	826.04	820.19
	YBT1520_13500	scwl7	N-acetylmuramoyl-L-alanine amidase	2120.57	7.13	495.06	578.49
	YBT1520_17375	xhlA3	cof family hydrolase	0	0	0	4214.51
	YBT1520_17380		transposase	0	0	0	0
	YBT1520_17385		transposase for insertion sequence element IS232	0	0	0	0
	YBT1520_17390		DNA replication protein	0	0	0	0
	YBT1520_17395		hypothetical protein	242.7	394.38	6.3	69.31
	YBT1520_17400		hypothetical protein	11.62	90.58	0	0
	YBT1520_17405		hypothetical protein	239.24	245.52	0	0
	YBT1520_17410		hypothetical protein	153.14	0	81.04	0
	YBT1520_17415		hypothetical protein	28.42	35.32	0	0
	YBT1520_17420		Syd protein	80.82	203.8	239.8	174.82
	YBT1520_17425		hypothetical protein	0	42.99	100.34	69
	YBT1520_17430		SMI1 / KNR4 family protein	674.3	519.33	721.98	625.73
	YBT1520_17435		hypothetical protein	11.27	31.26	1640.4	916.21
	YBT1520_17440		hypothetical protein	176.96	105.12	32.43	0
	YBT1520_17445		hypothetical protein	300.39	403.85	143.08	49.07
	YBT1520_17450		hypothetical protein	212.22	276.88	0	109.19
	YBT1520_17455		deoxyinosine 3'endonuclease	2056.91	2156.99	0	37.41
Urease synthesis gene operon	YBT1520_19325	ureT	high affinity nickel transporter protein	0	0	92.28	114.71
	YBT1520_19330	ureI	transporter	96.85	148.54	13.13	776.64
	YBT1520_19335	ureD	urease accessory protein UreD	564.04	621.95	1578.85	1885.18
	YBT1520_19340	ureG	urease accessory protein UreG	786.12	922.15	19.97	0
	YBT1520_19345	ureF	urease accessory protein UreF	431.87	520.69	70.78	0
	YBT1520_19350	ureE	urease accessory protein UreE	435.85	597.82	7.76	62.56
	YBT1520_19355	ureC	urease subunit alpha	603.71	1130.33	40.73	0
	YBT1520_19360	ureB	urease subunit beta	1326.67	1784.17	273.77	1415.56
	YBT1520_19365	ureA	urease subunit gamma	1973.94	2800.91	184.85	25.75
